# Update on biologic and small molecules treatments in pediatric allergic diseases

**DOI:** 10.1111/pai.70454

**Published:** 2026-09-25

**Authors:** Herman T. den Dekker, Amandine Divaret Chauveau, Benoit Sterling, Stéphanie Coopman, R. Sharon Chinthrajah, Stéphanie Lejeune, Dorothea Holter, Thomas Eiwegger, Stéphanie Mallet, Mariëlle W. Pijnenburg, Antoine Deschildre

**Affiliations:** ^1^ Department of Pediatrics/Pediatric Respiratory Medicine and Allergology, Erasmus MC – Sophia Children's Hospital University Medical Center Rotterdam Rotterdam the Netherlands; ^2^ Pediatric Allergy Unit, Children's Hospital University Hospital of Nancy Vandoeuvre les Nancy France; ^3^ UR 3450 DevAH, Campus Brabois Santé University of Lorraine Vandoeuvre les Nancy France; ^4^ Aix‐Marseille Univ APHM, Hôpital Timone, Service de Dermatologie‐Vénéréologie Marseille France; ^5^ Department of Pediatric Gastroenterology Hepatology and Nutrition, Centre Hospitalier Universitaire Lille Reference Center for Congenital Oesophageal Anomalies Lille France; ^6^ Sean N. Parker Center for Allergy and Asthma Research Stanford University Palo Alto California USA; ^7^ Univ. Lille, CHU de Lille, Service de Pneumologie et d'Allergologie Pédiatrique, Hôpital Jeanne de Flandre Lille France; ^8^ INSERM Unit 1019, CNRS UMR 9017, CHU Lille, Institut Pasteur de Lille, Center for Infection and Immunity of Lille Université de Lille Lille Cedex France; ^9^ Department of Pediatrics and Adolescent Medicine Karl Landsteiner University Krems Austria; ^10^ Department of Pediatric and Adolescent Medicine University Hospital St. Pölten St. Pölten Austria; ^11^ Translational Medicine Program, Research Institute Hospital for Sick Children Toronto Ontario Canada; ^12^ Department of Immunology University of Toronto, Temerty Faculty of Medicine Toronto Ontario Canada

**Keywords:** atopic dermatitis, biologics, children, chronic spontaneous urticaria, eosinophilic esophagitis, food allergy, severe asthma, small molecules

## Abstract

Biologics have revolutionized the treatment of severe allergic diseases in children that cannot be controlled by conventional treatments. These diseases are commonly driven by dysregulation of immune response, often driven by Type 2 pathways. By targeting them, biologics have the potential for treating one or more allergic diseases. Thus, a “treat to target” strategy is now prioritized, allowing treatment of allergic diseases in a personalized approach. The pioneer anti‐IgE monoclonal antibody, omalizumab, was initially indicated for the treatment of moderate to severe allergic asthma and is now indicated for chronic spontaneous urticaria (CSU), chronic rhinosinusitis with nasal polyps (CRSwNP), and food allergy (FA), highlighting the ability of biologics to target multiple allergic diseases. Other biologics have been developed in asthma and other allergic diseases, including mepolizumab (anti‐IL5), benralizumab (anti‐IL5 receptor), dupilumab (anti–IL‐4 receptor α), and more recently tezepelumab (anti‐TSLP). Clinical trials in children and/or teenagers have demonstrated their efficacy and safety, confirmed by real‐life studies, in asthma, atopic dermatitis (AD), CSU, and more recently in eosinophilic esophagitis (EoE) and FA. Beyond biologics, small molecules such as Janus kinase inhibitors target other key pathways and have already been approved in AD, with ongoing clinical trials in other allergic diseases. In the pediatric specific context, these treatments raise questions, particularly regarding the choice of biologics and the goals. In the long term, the sustained efficacy, safety and impact on the natural history of allergic diseases need to be further studied. The aim of this review is to discuss the most recent data in the use of biologics and small molecules in pediatric allergic diseases and to highlight the remaining gaps in knowledge.

## Graphical abstract

This graphical abstract summarizes key targets, mechanisms of action, and indications of biologic and small molecules treatments in pediatric allergic diseases.abbreviations : BAS, basophil; BTK, Bruton tyrosine kinase; cDC, conventional dendritic cell; IgE, immunoglobulin E; FCER1, high affinity IgE receptor; IL, interleukin; IL4R, IL4 receptor; JAK, Janus kinase; MC, mast cell; PC, IgE producing plasma cell; ILC2, group 2 innate lymphoid cell; TSLP, thymic stromal lymphopoietin.

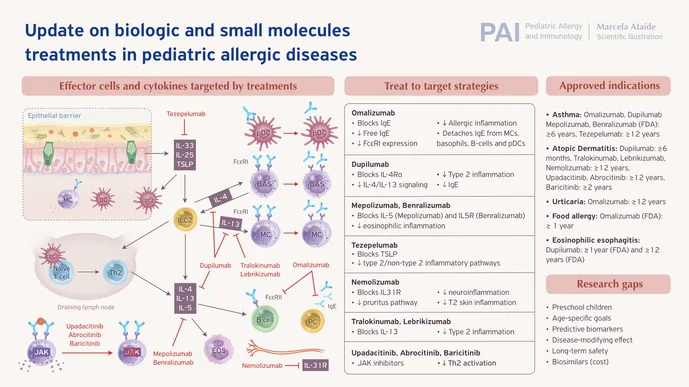

**Key message**

Biologics and small molecule treatments are reshaping the management of pediatric allergic diseases through targeted and personalized treatment strategies. Their efficacy and safety have been demonstrated for different pediatric allergic diseases. However, unmet needs remain for some of them including preschool children. Future will rely on clinical trials considering age group specificities, better biomarker‐guided treatment choice, and improved global access to these treatments.

## 1 INTRODUCTION

The prevalence of allergic diseases, including asthma, rhinitis, atopic dermatitis (AD), food allergy (FA), and eosinophilic gastrointestinal disorders (EGIDs), has increased over recent decades.^1^ These conditions frequently coexist, supporting the concept of allergic multimorbidity driven by shared pathophysiological mechanisms rather than the classical atopic march.^2^ Advances in the understanding of the allergic cascade and inflammatory pathways, particularly the role of epithelial barrier dysfunction, have enabled the identification of therapeutic targets for biologics and small molecules.^3^, ^4^ Since the approval of omalizumab (OMZ) more than 20 years ago for moderate [Food and Drug Administration (FDA)] and severe asthma [FDA, European Medical Agency (EMA)], biologics have expanded across multiple allergic diseases and indications continue to grow.^5^, ^6^ In parallel, small molecules—including Janus kinase (JAK), Bruton Tyrosine kinase (BTK), and PDE4 inhibitors—target additional signaling pathways involved in allergic inflammation. Despite pediatric trials studying these drugs, evidence remains limited for several age groups (e.g., preschoolers and school‐age children) and indications, and pediatric‐specific treatment goals are not always addressed.

Here, we review recent advances in biologics and small molecules for pediatric allergic diseases. We first outline key mechanistic pathways underlying allergic inflammation and the development of targeted therapies, including the concept of “treat‐to‐target” strategies. We then summarize recent data in asthma, a pioneering indication, as well as AD/chronic spontaneous urticaria (CSU), FA, and EGIDs, and highlight remaining knowledge gaps.

## 2 OVERVIEW ON BIOLOGICS IN PEDIATRIC ALLERGIC DISEASES

### 2.1 Common pathways driving allergic diseases

**FIGURE 1:**
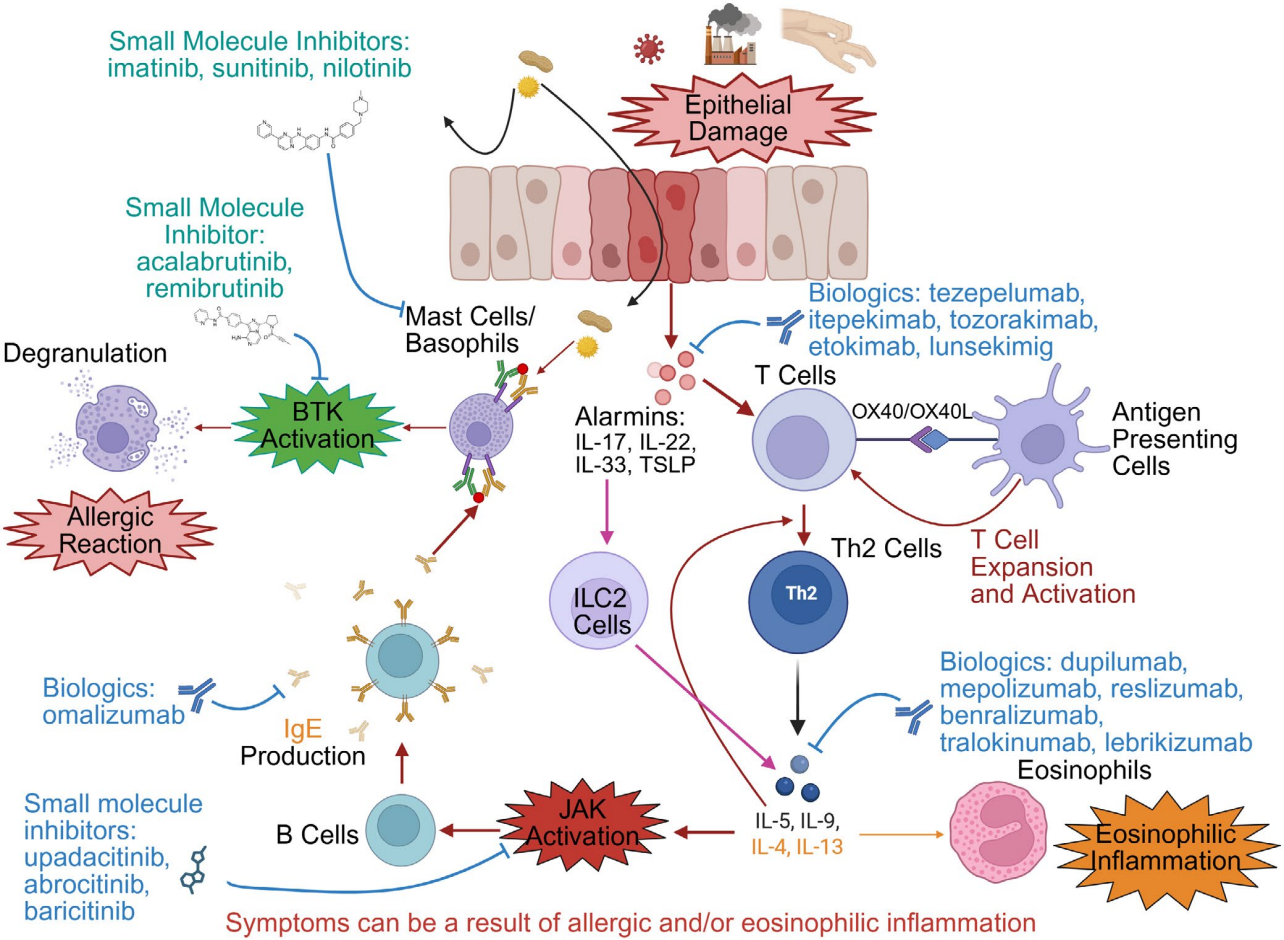
Biologics and small molecules treatments: Targeting the allergic pathway in allergic diseases.

Epithelial barrier disruption induces the release of alarmins such as thymic stromal lymphopoietin (TSLP), interleukin (IL) 33, IL‐33, and IL‐25.^7^, ^8^ These cytokines promote differentiation of naïve T cells into Th2 cells within a permissive genetic and inflammatory environment. These alarmins also directly activate ILC2s, innate lymphoid cells (ILC) that rapidly produce IL‐5 and IL‐13 to amplify early type 2 inflammation independently of antigen‐specific T cell activation.^9^ Th2 responses are characterized by the production of IL‐4, IL‐5, and IL‐13 (Figure 1). IL‐4 promotes Th2 polarization and B‐cell class switching to IgE, which binds high‐affinity receptors on mast cells and basophils, enabling immediate hypersensitivity reactions. IL‐5 regulates eosinophil differentiation, activation, survival, and recruitment, contributing to tissue damage in conditions such as asthma, EGIDs, and chronic rhinosinusitis with nasal polyps (CRSwNP). IL‐13 shares receptor components with IL‐4 and contributes to IgE production but primarily drives goblet cell hyperplasia, airway smooth muscle contraction, fibrosis, and mucus production. Activation of the Th2 pathway culminates in IgE‐mediated mast cell degranulation and release of mediators including histamine, producing allergic symptoms. Leukotrienes and prostaglandins further sustain inflammation. Chronic Th2‐driven inflammation leads to eosinophilia, epithelial damage, fibrosis, and structural remodeling that characterizes severe allergic disease. Beyond biologics, small molecule inhibitors represent a growing therapeutic class that targets intracellular signaling cascades, including JAK, BTK, and phosphodiesterase 4 (PDE4) inhibitors.^10^

### 2.2 Targeting allergic pathways

Recent approaches have focused on the potential of using biologics and small molecule inhibitors to directly target the shared allergic pathways (Figure 1). Unlike antigen‐specific approaches, these strategies may provide protection against multiple allergens or triggers. The concept of allergic multimorbidity further supports targeting common inflammatory pathways through treat‐to‐target approaches addressing coexisting diseases.^2^ Accordingly, several biologics and small molecules are now approved by EMA/FDA for multiple allergic diseases, enabling simultaneous treatment of associated conditions, as illustrated by OMZ in asthma and FA.^11^, ^12^, ^13^, ^14^

Anti‐IL‐5 agents are approved for eosinophilic asthma among other conditions: mepolizumab [CRSwNP (adults), eosinophilic granulomatosis with polyangiitis (adults), hypereosinophilic syndrome (≥12 years), and eosinophilic chronic obstructive pulmonary disease (COPD) (adults)], reslizumab, and benralizumab [eosinophilic granulomatosis with polyangiitis]. Dupilumab targets the shared IL‐4rα receptor, blocking IL‐4 and IL‐13 signaling, and is EMA/FDA‐approved for AD (≥6 months), eosinophilic or corticosteroid‐dependent asthma (≥6 years), CRSwNP (adults), COPD (adults), and eosinophilic esophagitis (EoE) (≥6 years). Emerging therapies target upstream mediators. Tezepelumab (anti‐TSLP) is approved for severe asthma (≥12 years) and CRSwNP (adults). Others are under investigation. The Anti‐IL‐33 agents are not currently approved but itepekimab, tozorakimab, and etokimab are being evaluated for asthma, COPD, and AD. Other approaches target inflammatory cytokines such as IL‐17 (secukinumab, ixekizumab, brodalumab, bimekizumab; FDA‐approved for psoriasis) and IL‐23 (risankizumab‐rzaa, guselkumab, tildrakizumab‐asmn, mirikizumab‐mrkz; approved for psoriasis or ulcerative colitis). Nemolizumab targets IL‐31 and is EMA/FDA‐approved (≥12 years) for AD and prurigo nodularis. Small molecules such JAK inhibitors—upadacitinib, abrocitinib (≥12 years), baricitinib (≥2 years) are already approved in AD. Bruton's Tyrosine Kinase (BTK) mediates signaling downstream of FcεRI which forms a complex with IgE to bind to allergens. Early studies suggest that BTK inhibitors such as acalabrutinib may represent promising therapeutic options in allergic diseases.^15^, ^16^

### 2.3 Biosimilars

Biologics are expensive and may range from 30.000 to 40.000 USD per patient per year. Biosimilars are biologic medications with chemical properties highly similar to already approved biologics and are typically more affordable. In clinical practice, biosimilars could significantly reduce the economic burden of biologic treatments, increasing their availability, not only in resource‐constrained settings but also in prosperous countries.^17^ The first biologic for which a biosimilar is on the market for allergic diseases is OMZ. Recent studies have shown that an OMZ biosimilar is non‐inferior in terms of efficacy, safety, and immunogenicity.^18^, ^19^ However, careful monitoring for potential differences in pharmacokinetics or immunogenicity remains important, especially in children in whom safety profiles may differ from those in adults.

## 3 ASTHMA: WHAT IS NEW IN THIS PIONEERING INDICATION?

### 3.1 Biologics in pediatric severe asthma

The use of biologics in pediatric SA has evolved significantly with numerous trials and real‐world studies contributing to our understanding of their efficacy and safety (Table 1).^20^, ^21^, ^22^, ^23^, ^24^, ^25^, ^26^, ^27^, ^28^, ^29^, ^30^, ^31^, ^32^, ^33^, ^34^, ^35^, ^36^, ^37^, ^38^, ^39^, ^40^, ^41^, ^42^, ^43^, ^44^, ^45^, ^46^, ^47^, ^48^ Currently, four biologics have been approved by EMA for use in children and/or adolescents with SA: OMZ, mepolizumab, dupilumab, tezepelumab; the FDA approved a fifth biologic, benralizumab, for patients ≥6 years. OMZ has consistently been shown to reduce exacerbations and inhaled corticosteroids (ICS) dose in children with allergic asthma, but improvement of control and lung function was only reported in real‐world studies.^20^, ^21^, ^22^, ^23^, ^24^, ^25^, ^26^, ^27^, ^28^, ^29^, ^30^, ^31^, ^32^, ^33^, ^34^, ^35^, ^49^ Mepolizumab reduces exacerbations in children with eosinophilic asthma, although non‐response can be observed.^36^, ^37^, ^38^, ^50^ This differential clinical response has been associated with specific eosinophil‐ and epithelial‐associated molecular pathways as identified by airway transcriptomics.^38^ Dupilumab showed significant reductions of exacerbations, improved lung function and enhanced asthma control in children ≥6 years.^39^, ^40^, ^41^, ^42^, ^43^, ^44^, ^45^, ^46^, ^51^ Limited data are available on the efficacy of Tezepelumab. In the study by Menzies‐Gow et al., only 82 adolescents were included and the difference in exacerbations was not significant in this age group.^47^

**TABLE 1:** Main clinical trials and real‐world studies on biologics in school‐aged children and adolescents with severe asthma.

Study design	Population	Indications	Treatment (dose, frequency)	Efficacy	Tolerance
Omalizumab					
Milgrom et al. *Pediatrics* 2001^20^ Randomized double‐blind placebo‐controlled trial (RDBPCT) (0) Screening phase: 1 week (1) Initiation phase 4–6 weeks: Switch ICS to beclomethasone dipropionate (BDP), adjusted doses (2) ICS maintenance dose phase (16 weeks) (3) ICS dose reduction phase (12 weeks) (minimum effective doses) 4/Open‐label trial Lemanske et al. Pediatrics 2002^21^ Study focused on QoL Silkoff et al. *Pediatrics* 2004^22^ Supplementary study in two centres, focusing on FeNO variation	334 patients aged 6–12 years (69% boys), 225 OMZ 109 Placebo (ratio 2:1) 29 patients 18 OMZ 11 Placebo	Allergic asthma well‐controlled by doses of ICS (168–420 mg/day BDP) and SABA on demand	OMZ Subcutaneous 0.016 mg/kg/IgE [IU/mL] Every 4 weeks	1/Reduction in BDP doses: 100% OMZ vs. 67% placebo (*p* = .001) 2/Reduction in asthma attacks (phase 3): 18% OMZ vs. 39% Placebo (*p* < .001) 3/Reduction in average duration of attacks: 0.42 vs. 2.72, *p* < .001 4/Reduction in SABA use: 0 puffs vs. 0.46 (*p* = .004) 5/Reduction in average number of days out of school, number of visits for asthma 6/No differences in asthma symptom scores/FEV data (FEV, FVC, PEF) At the end of reduction phase (3), in OMZ patients: Significant improvements in QoL scores (global, “activities” and “symptoms” domains) More clinically relevant changes (>0.5 points) in QoL scores During the maintenance phase (2): No significant difference in FeNO variation from baseline between the OMZ and placebo groups Analysis over 52 weeks: Significant reduction in FeNO compared to baseline in the OMZ group, despite a decrease in corticosteroid doses	No serious AEs Frequency and types of all AEs similar in both groups Majority of AE mild to moderate in severity No AEs suggesting immune complex formation Urticaria reported in 9 patients on OMZ (4%) vs. 1 patient on placebo (0.9%)
Lanier et al. *JACI* 2009^23^ RDBPCT (0) 1 week selection phase (1) Screening phase 8S: dose adjustment over 4 weeks (2) After randomization, treatment period without dose adjustment of ICS: 24 weeks (3) Treatment period with dose adjustment of ICS: 28 weeks Kulus et al. *Curr Med Res Opinion* 2010^24^	627 patients aged 6–12 years (68% boys), 421 OMZ 206 Placebo (ratio 2:1) Efficacy analysis in 384 OMZ, 192 Placebo Analysis in 235 OMZ 159 Placebo	Moderate to severe persistent allergic asthma inadequately controlled by medium or high doses of ICS + − other control medication Subgroup not controlled by high doses of ICS (500 μg/day fluticasone) + LABA, + − other	OMZ Subcutaneous 75–375 mg 1–2 times/month	During the 24‐week phase (2): Reduction in the rate of clinically significant attacks (worsening of symptoms requiring doubling of ICS doses and/or systemic corticosteroid therapy) in the OMZ group: 0.45 vs. 0.64; rate R 0.69; *p* = .007 Over the 52‐week period (2 + 3): Crisis rate in the OMZ group reduced by 43% compared to placebo (*p* < .001) Reduction in severe attacks In the OMZ group: 34% reduction in clinically significant seizure rate compared to placebo over 24 weeks (0.42 vs. 0.63, rate ratio 0.662; *p* = .047) and 50% reduction over 52 weeks (*p* < .001)	Acceptable safety profile, no difference in the occurrence of AEs
Busse et al. *N Engl J Med* 2011^25^ ICATA: Randomized, DBPC trial Multicentre, urban setting (1) 4 weeks screening phase (2) 60‐week treatment phase	419 patients Ages 6–20 IgE: 30–1300 kU/L	Moderate to severe allergic asthma: 73%	OMZ Subcutaneous 0.016 mg/kg/IgE every 2 or 4 weeks	OMZ vs. Placebo: Number of days with asthma symptoms: 25.5% % of patients experiencing at least one attack: −38%.	≥1 severe AE: 13.7% (placebo) vs. 6.3% (OMZ), (*p* = .02), OMZ: gastrointestinal disorders > hematological disorders, 1 case of anaphylaxis (cough, pharyngeal pruritus)
Teach et al. *J Allergy Clin Immunol* 2015^26^ PROSE: RDBPCT, multicentre, urban, Inclusions Nov‐March Intervention phase: 4–6 weeks before the start of the school year date – 90 days after 3 arms: (1) OMZ; (2) Boost ICS 4–6 weeks (≤1000 μg/day fluticasone equivalent) before the start of the school year; (3) Placebo	478 children (aged 6–17) At least one recent attack (previous 19 months)	Allergic asthma Variable severity 200 μg/day fluticasone equivalent 184 (35%) receive step 5 treatment	OMZ Subcutaneous 75–375 mg 1–2 times/month	Probability of experiencing at least one seizure: 11.3% in the OMZ group vs. 21.0% in the placebo group; OR: 0.48 (95% CI, 0.25 to 0.92) After stratification by tier: Persistent effect of OMZ vs. placebo in patients at tier 5 No OMZ vs. ICS boost effect in patients at stages 2–4: 8.4% vs. 11.1%; OR, 0.73 (95% CI, 0.33–1.64) Benefit linked to improved antiviral defenses (interferon response)	≥1 AE: No difference between OMZ and placebo, regardless of stratification by stage Grade 1 anaphylaxis: 3 in the OMZ arm 3 in the ICS boost arm 2 in the placebo arm No severe AE in the OMZ arm
Brodlie et al. *Arch Dis Child* 2012^27^ Open‐label therapeutic trial OMZ for 16 weeks	34 children (5–16 years old, 15 children <12 years old) IgE: 30–1300 kU/L Median IgE: 411 kU/L	Severe asthma under long‐term systemic corticosteroid therapy (3 months)	OMZ Subcutaneous 75–375 mg according to the dosage table, every 2 or 4 weeks	Median daily dose of prednisolone reduced from 20 mg (2.5–50 mg) to 5 mg (0–40 mg) (*p* < .0001) 7 children stopped prednisolone completely The dose was reduced in 29 children, with a median reduction of 15 mg (2–40 mg) Subgroup analysis in children <12 years of age: median dose reduction from 20 mg (5–50 mg) to 5 mg (0–40 mg) (*p* < .0001) QoL: improvement in median mini‐AQLQ score from 3.5 (1–8.4) to 5.9 (3.2–9.9) (*p* < .0001)	No major side effects reported (no details)
Deschildre et al. *Eur Respir J* 2013^28^ Prospective multicentre study (French tertiary centres) Progression at 1 year in children starting OMZ biotherapy Deschildre, et al. *Eur Respir J* 2015^29^ Same population Progression at 2 years in children starting OMZ biotherapy	104 6–18 years 47 6–12 years Median IgE: 1125 kU/L Polysensitisation (≥3 allergens): 66% Exacerbators (4.4/year) 78 children treated for 2 years	Severe allergic asthma resistant to high‐dose treatment (median dose 703 μg/day fluticasone equivalent)	OMZ Subcutaneous 75–375 mg according to the dosage table, every 2 or 4 weeks	Improvement in control: good control: from 0% at initiation to 53% at week 20% and 67% at week 52 Rate of attacks: −72% Hospitalization rate: −88.5% FEV1: +4.9% Daily ICS doses: −30%. Improvement in control: good control: 80% at week 104 Attack rate: −83%. No hospitalisations No significant additional gain in FEV1 or average daily dose of ICS	First year of treatment: ≥1 AE: 47 patients (46%) Pain at the injection site: 26% 6 patients with severe AE leading to discontinuation of treatment: multisystemic reaction (4), generalized urticaria (1), anaphylaxis (1) 1 case of anaphylaxis unrelated to treatment (kiwi allergy) Second year of treatment: 8 patients with AE leading to discontinuation of treatment: fatigue (6) and multisystemic reaction (2)
Lieberman et al. *J Allergy Clin Immunol* 2016^30^ Case–control study 30 cases of anaphylaxis associated with OMZ/88 OMZ controls without anaphylaxis		Severe allergic asthma resistant to high‐dose treatment	OMZ Subcutaneous 75–375 mg according to the dosage table, every 2 or 4 weeks		80% of cases (24/30): cutaneous‐mucosal and respiratory symptoms 70% of events within 1 h of injection 39% with doses 1–3, 29% with doses 4–20 History of anaphylaxis from any cause: risk factor for anaphylaxis with OMZ (OR, 8.1; 95% CI, 2.7–24.3).
Pitrez et al. *Pediatr Pulmonol* 2017^31^ Brazilian retrospective study	14 children (mean age: 11.9 years)	Severe allergic asthma resistant to high‐dose treatment, including systemic CS	OMZ Subcutaneous 75–375 mg according to the dosage table, every 2 or 4 weeks	Hospitalization rate reduced by 70%. Discontinuation of oral corticosteroid therapy: 8 children/9	
Licari et al. *Curr Respir Med Rev* 2017^32^ Italian real‐life experience: 1‐year multicenter (13 tertiary care centers) observational study conducted in children and adolescents with severe allergic asthma	47 patients (27 < 12 years)	Confirmed severe allergic asthma	OMZ Subcutaneous 75–375 mg according to the dosage table, every 2 or 4 weeks	Reduction in the number of asthma exacerbations: 1.03 vs. 7.2 after 6 months (*p* < .001) and 0.8 after 12 months (*p* < .001) Reduction in hospital admissions by 96%. At 12 months, improvement in FEV1: 79% PV at baseline, vs. 90% PV at 6 months, 91% PV at 12 months Corticosteroid sparing effect	No serious AE: *n* = 1 asthenia after the first administrations; *n* = 1 hyperaemia and itching of chest and arms, reversible after antihistamine therapy; *n* = 1 mild local reaction in the injection site
Humbert et al. *Eur Respir J* 2018^33^ STELLAIR study French retrospective multicentre real‐world study Response to OMZ based on blood eosinophilia before treatment Good response to OMZ at 4–6 months: ^1^Assessment by the physician (GETE scale)^2^; ≥40% reduction in annual attack rate ^3^ Combined response^1^ +^2^	872 patients with severe allergic asthma including 149 children aged 6–17 years Blood eosinophilia ≥300/mm^3^ in 73.8% of children	Severe allergic asthma resistant to high‐dose treatment	OMZ Subcutaneous 75–375 mg according to the dosage table, every 2 or 4 weeks	According to^1^ assessment by the physician (GETE scale): 67.2% of adults and 77.2% of children were good responders According to^2^ reduction in attack rate: 71.1% of adults and 78.5% of children were good responders According to^3^ combined response: 58.2% of adults and 67.8% of children were good responders In children: 70.9% good responders if eo ≥300/mm^3^ (*n* = 110) vs. 59% if eo <300/mm^3^ (*n* = 39) 72.5% good responders if eo ≥600/mm^3^ (*n* = 80) vs. 62.3% if eo <600/mm^3^ (*n* = 69)	
Sesé et al. *Clin Exp Allergy*. 2019^34^ Children selected from the Trousseau Severe Asthma Programme cohort Single‐centre cohort of children with severe asthma Cluster analysis to identify predictive markers of good response in the subpopulation of children with SA receiving OMZ for at least 4 months	45 children aged 6–18 years	Severe allergic asthma resistant to high‐dose treatment Poor responders: ^1^ ACT <20 (2) Persistence of severe attacks ^3^ No improvement in respiratory function	OMZ Subcutaneous 75–375 mg according to the dosage table, every 2 or 4 weeks	Cluster 1: 16 children with eosinophilic asthma (eo >300/mm^3^, *n* = 15 (94%), *p* = .001), impaired respiratory function (mean FEV1 65% + −10 PV, *p* = .006) and poor response to OMZ (*n* = 1 (6%) good responder, *p* < .001); Cluster 2: 11 children with environmental tobacco smoke exposure (*n* = 6 (55%), *p* = .016), normal respiratory function, inconsistent response to OMZ (*n* = 4 (37%) good responders); Cluster 3: 18 children with multiple allergic comorbidities (*n* = 15 (83.3%), *p* = .001), including atopic dermatitis (*n* = 13 (72%), *p* < .001), polysensitization (*n* = 15 (83%) *p* < .009), normal respiratory function and good responders to OMZ (*n* = 16 (89%) good responders, *p* < .001).	
Nieto‐Garcia et al. *Ped Allergy Immunol* 2021^35^ ANCHORS: multicenter, observational, retrospective cohort study conducted in 25 Pediatric Allergy and Pulmonology units in Spain, up to 6 years of follow‐up	484 children, mean age: 11.1 years	Severe persistent allergic asthma, high‐dose ICS + controllers	OMZ Subcutaneous 75–375 mg according to the dosage table, every 2 or 4 weeks	Sustained exacerbation reduction: (−80.2% on the first year) sustained up to year 6 Improved control: 8.4% controlled at baseline, 45.0% at year 1, 89.3% at year 6	Long‐term safety consistent with known profile. 4.3% with at least one AE: headache, unspecific symptoms like malaise, fatigue, asthenia, low‐grade fever, myalgia, and/or flu‐like syndrome, injection site pain/reaction, dizziness/loss of consciousness/vasovagal syncope, transient urticaria, anaphylaxis.
Mepolizumab (MPZ)					
Yancey et al. *Allergy Asthma Clin Immunol* 2019^36^ Post‐hoc analysis of adolescents included in Phase II/III Mepolizumab trials (MENSA, MUSCA, DREAM, SIRIUS)	37 adolescents 12–17 years MENSA (*n* = 25), MUSCA (*n* = 9) DREAM (*n* = 1) SIRIUS (*n* = 2)	Severe eosinophilic asthma	MENSA: placebo or 75 mg IV MPZ of 100 mg SC MPZ MUSCA/SIRIUS: placebo or 100 mg MPZ DREAM: Placebo or 75, 250, or 750 mg IV MPZ	40% reduction in the annual rate of clinically significant exacerbations Reduction in blood eo counts in response to MPZ in adolescent patients	Good safety profile, similar to adults Most common AEs: headache, nasopharyngitis, and upper abdominal pain
Gupta et al. *J Allergy Clin Immunol* 2019^37^ Open‐label, uncontrolled trial 2 phases: A/12 weeks treatment B/52 weeks treatment	36 children aged 6–11 years (mean: 8.6 years) 30 children (83%) in phase B	Eosinophilic asthma At least 2 attacks per year resistant to treatment (>200 μg/day fluticasone equivalent +2nd controller medication) Eo >150/mm^3^ or Eo >300/mm^3^ during the year	Mepolizumab Subcutaneous 40 mg (<40 kg) 100 mg (≥40 kg) Every 4 weeks	Improvement in control: Average ACT of 17.6 at inclusion to 22.0 at 36 weeks and 22.0 at 52 weeks 69% reduction in seizure rate: 14/30 (46%): at least 1 seizure at week 52, including 5 requiring emergency room visits/hospitalization Average annualized seizure rate of 3.5 exa/year at inclusion to 1.09 exa/year at 52 weeks Reduction in mean eo rate from 336/mm^3^ at inclusion to 47/mm^3^ at 52 weeks	At 52 weeks: 27/30 AEs: bronchitis (9), headaches (8), asthma attacks (7) 8 treatment‐related AEs: headache (4), abdominal pain (3), fever (2) 7/30 severe AEs including 1 anaphylaxis (FA/peanut) No severe AEs related to treatment At 12 weeks: 2/36 patients with anti‐mepolizumab antibodies (1 patient not included in phase B) At 52 weeks: No detection of anti‐mepolizumab neutralizing antibodies
Jackson, *Lancet*. 2022^38^ MUPPITS‐2: RDBPCT 9 urban centres in the USA ≥2 attacks in the previous year Blood eosinophils count ≥150/mm^3^ 1:1 randomization 52 weeks	335 6–17A MPZ (*n* = 146) Placebo (*n* = 144)	Severe asthma ≥2 attacks per year PNN ≥150/mm^3^ GINA steps 3, 4 or 5: ICS ≥250 μg/day fluticasone	MPZ: 6–11 years: 40 mg/4 weeks	Decrease in attacks in the MPZ group: rate ratio 0.73; 0.56–0.96; *p* = .027	
Dupilumab (DPM)					
Castro et al. *N Engl J Med* 2018^39^ LIBERTY ASTHMA QUEST 12 weeks, 52 weeks Maspero et al. *Allergy*. 2021^40^ LIBERTY ASTHMA QUEST post hoc analysis assessing the efficacy of dupilumab in adolescents (12–17 years) compared with adults	1902 patients ≥12 years Post‐hoc analysis on the 107 adolescents	Uncontrolled moderate to severe asthma/ICS (fluticasone eq ≥500 μg/day) + 1–2 additional controllers; FEV1 ≤80% PV (≤90% PV in 12–17 years); FEV1 reversibility; ACQ‐5 ≥ 1.5; ≥ 1 severe exa in previous y	200 mg every 2 weeks (*n* = 34) vs. matched Placebo (*n* = 21) 300 mg every 2 weeks (*n* = 34) vs. matched Placebo (*n* = 18)	No significant difference in severe exacerbations in the adolescents subgroup, unlike the results observed in adults. Significant improvement in FEV1 at 12 weeks, maintained at 52 weeks: +0.36 L (95% CI: 0.12–0.61) for the 200 mg dose and +0.27 L (95% CI: 0.02–0.52) for the 300 mg dose. More effective in cases of blood eo ≥300/mm^3^ and/or FeNO ≥50 ppb	Well tolerated AE: injection‐site reactions eosinophilia No difference in safety data between adolescents and adults
Bacharier, *N Engl J Med* 2021^41^ VOYAGE: RDBPCT Multicentre, multinational 52 weeks Fiocchi, *Eur Respir J* 2023^42^ Papadopoulos et al. *Allergy* 2023^43^ Guilbert et al. *J Asthma Allergy* 2024^44^	408 children aged 6–11 years 273 DPM 135 Placebo 2 patient profiles: (1) T2 phenotype (blood eo ≥150/mm^3^ and/or FeNO ≥20 ppb): 236 DPM/114 Placebo (2) Phenotype eo ≥300/mm^3^ 175 DPM/84 Placebo	Moderate to severe persistent asthma inadequately controlled with medium or high doses of ICS + − other control medication At least 1 severe attack in the past year FEV1 ≤ 95% PV Reversibility FEV1 ≥ 10%	DPM Subcutaneous 100 mg (≤30 kg) 200 mg (>30 kg) Every 2 weeks	At 52 weeks: (1) In patients with T2 phenotype: Annualized exacerbation rate of 0.31 (0.22–0.42) Dupilumab vs. 0.75 (0.54–1.03) Placebo (*p* < .001) Change in pre‐inclusion FEV1: 10.5% + −1.01 Dupilumab vs. 5.3% + −1.4 Placebo (*p* < .001) Improvement in control (2) In patients with eo phenotype ≥300/mm^3^: Annualized exacerbation rate of 0.24 (0.16–0.35) Dupilumab vs. 0.67 (0.47–0.95) Placebo (*p* < .001) Improvement in pre‐inclusion FEV1 Improved control	Frequency of AEs comparable between the Dupilumab group (83%) and the Placebo group (79.9%) Treatment discontinuation due to an AE: 1.8% Dupilumab vs. 1.5% Placebo Severe AEs in 13 patients (4.8%) Dupilumab vs. 6 patients (4.5%) Placebo Eosinophilia in 5.9% Dupilumab vs. 0.7% Placebo Non‐severe parasitic infections in 7 patients (2.6%) Dupilumab vs. 0 Placebo
Shi et al. *BMC Pulm Med* 2024^45^ Real‐world data from 2 Chinese centres, including children treated with dupilumab for at least 24 weeks	25 children aged 6–13 y	Severe asthma based on GINA criteria	Dupilumab every 2–4 weeks for 24 weeks in a weight‐tiered fashion according to the dupilumab instruction in China	After 24 weeks: Decrease in mean number of severe asthma exacerbation: 0.16 ± 0.37 vs. 1.44 ± 0.58, *p* = .00 Improvement in ACT score: 24.4 ± 0.8 vs. 18.3 ± 0.8, *p* = .00; and in pACT score: 26.0 ± 1.2 vs. 18.6 ± 1.0, *p* = .00 Inprovement in FEV1 (92.85 ± 11.21 vs. 82.03 ± 15.05, *p* = .01) and PEF25‐75 Decrease of Patient‐Oriented Eczema Measure (POEM) and Peak Pruritus Numerical Rating Scale (NRS), Rhinitis Four‐point, and Rhinitis Visual Analogue Scale (VAS) scores	1 patient with injection‐site reaction
Wanin et al. *Allergy* 2026^46^ PEDIASTHMA: 52‐week, multicenter, observational, prospective, retrospective, real‐world study conducted at 15 sites across metropolitan France	Description of the first 50 adolescents (66% male) After a protocol amendment (2022), children aged 6–11 y also included	Severe uncontrolled asthma despite high‐dose ICS‐LABA or requiring high‐dose ICS‐LABA to prevent it from becoming uncontrolled		Results at 52 weeks: pending	
Tezepelumab (TZP)					
Menzies‐Gow et al. *New Engl J Med* 2021^47^ NAVIGATOR Phase 3, multicenter, RDBPCT. 52 weeks	Patients ≥12–80 years 1061 patients (529 TZP, 532 Placebo) no age‐stratified efficacy or safety results reported	Severe Uncontrolled asthma: ICS (fluticasone eq ≥500 μg/day) +≥1 controller; FEV1 ≤80% PV (≤90% PV in 12–17 years); FEV1 reversibility; ≥2 severe exa in previous y 2 phenotypes based on baseline blood eo count > or ≤300 cells/microliter	Tezepelumab (210 mg) or placebo subcutaneously every 4 weeks	Decrease in annualized rate of asthma exacerbations: 0.93 (95% CI, 0.80–1.07) with TZP vs. 2.10 (95% CI, 1.84 to 2.39) with placebo (*p* < .001). Improved preBD FEV_1_ Improved PROMs: ACQ‐6, AQLQ, Asthma Symptom Diary (ASD)	Good safety profile, no difference between TZP and Placebo groups in terms of AE
Real‐World Multi‐Biologics Registries					
Liu et al. *ERJ Open Res* 2025^48^ Severe Pediatric Asthma Collaborative in Europe (SPACE, Clinical research collaboration of the ERS)	Multinational real‐world cohort of 250 children and adolescents (6–17 years) with severe asthma already on biologics: 56.8% OMZ 21.6% MPZ 21.6% DPM	Severe asthma: necessitating medium‐ to high‐dose ICS + a second controller (GINA step 4 and 5) but remained uncontrolled (frequent exacerbations and/or chronic asthma symptoms) despite this therapy.			

Abbreviations: ACQ, Asthma Control Questionnaire; AE, adverse event; AQLQ, Pediatric Asthma QoL Questionnaires; BDP, beclomethasone dipropionate; DPM, Dupilumab; FeNO, fractional exhaled nitric oxide; FEV1, forced expiratory volume in 1 s; FVC, forced vital capacity; GETE, Global Evaluation of Treatment Effectiveness; ICS, inhaled corticosteroids; LABA, Long‐acting beta agonists; MPZ, Mepolizumab; OMZ, omalizumab; PEF, peak expiratory flow; QoL, QoL; PV, predicted value; RDBPCT, Randomized double‐blind placebo‐controlled trial; SABA, short‐acting bronchodilators; TZP, Tezepelumab.

Safety data indicate that biologics are well tolerated and safe although needle fear may be a serious problem in (young) children and longer‐term safety data is needed, especially for children whose immune system is still developing.^52^ Common side effects include upper respiratory infections, headaches, injection site reactions, pyrexia, and urticaria.^30^, ^53^ Additionally, less common side effects in dupilumab treatment are high hypereosinophilia (defined as eosinophils >3000 per μL) in up to 5.9% of patients and conjunctivitis (especially if associated with AD) in up to 4%.^41^, ^54^

### 3.2 Measuring the response to biologics in pediatric severe asthma

Recently, the COMSA study (Core Outcome Measures sets for pediatric and adult Severe Asthma) aimed to develop a core outcome set for severe asthma.^55^ With a multi‐stakeholder consensus process, including patients and parents, Khaleva et al. identified five core outcomes in children and adolescents with SA: severe exacerbations (at least 3 days of systemic corticosteroids), asthma control (measured with the (Childhood) Asthma Control Test), quality of life (QoL) (measured with the Pediatric Asthma Quality of Life questionnaire), FEV1, and maintenance oral corticosteroid use. In addition to enabling defining response to biologic treatment in individual children, such a core outcome set may also facilitate comparison of different biologics in clinical trials. In a second study, the results of the COMSA study were taken further by developing a composite score with five levels of change for each outcome (CompOsite iNdexes For Response in asthMa, CONFiRM), leading to a score ranging from −31 (deterioration) to +69 (best possible response),^56^ with good discriminative ability for the response to biologics and excellent external validity. The value of this score in the real world has still to be shown.

### 3.3 Issues of choosing and stopping

**FIGURE 2:**
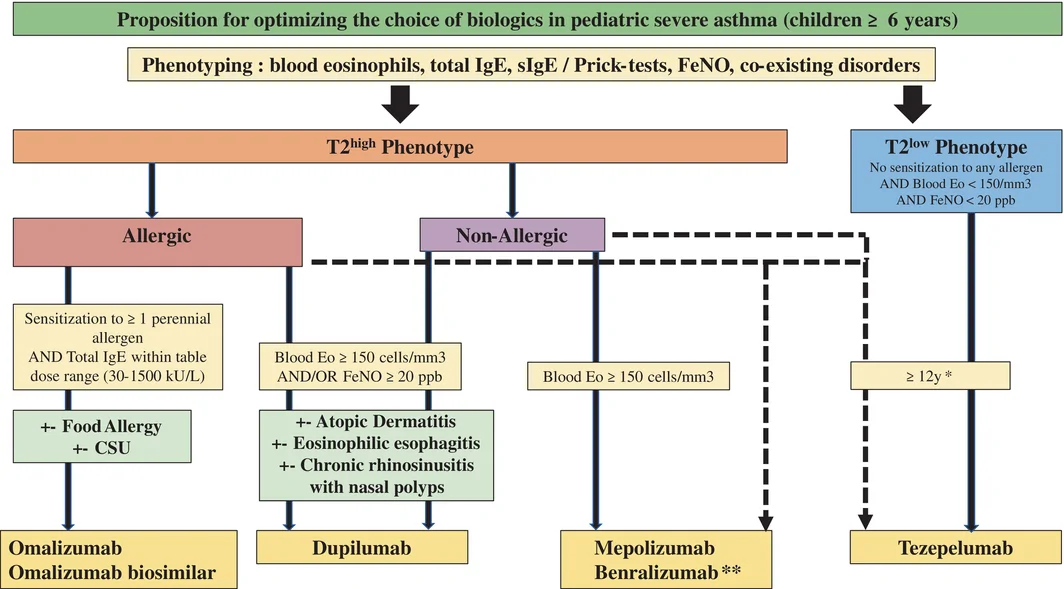
Proposition for optimizing the choice of biologics in pediatric severe asthma (Adapted from Lejeune et al.).^58^. sIgE, specific IgE; FeNO, fractional exhaled nitric oxide; ppb, parts per billion; T2, type 2; CSU, chronic spontaneous urticaria. Dotted arrows are considered as second choice; *Children aged 6–11 years with T2 low phenotypes are not eligible to any biologic. ** Benralizumab is FDA approved in children >= 6 years, EMA approved in children >=12 years

The selection of a biologic depends largely on asthma phenotype, biomarkers, and atopic comorbidities. For example, OMZ is typically prescribed for patients with severe allergic asthma triggered by allergens and with elevated IgE within the dosing table, while mepolizumab is more suited for eosinophilic asthma and dupilumab is most effective in patients with high FeNO and high eosinophils.^57^ Tezepelumab might be the only option in T2 low inflammation, although response is better in patients with high eosinophils/high FeNO.^47^ Co‐morbidities may be a key factor in choosing biologics in children and adolescents with severe asthma. In children with co‐morbid AD, eosinophilic esophagitis, or nasal polyps, dupilumab is a logical choice, while concomitant FA or CSU may drive the choice to OMZ. The latest French guidelines for the management of severe asthma feature an algorithm to guide the choice of biologics based on both patient phenotype and comorbidities.^58^ Here, we propose a set of criteria designed to optimize the choice in severe asthma (Figure 2). Other factors such as patient age, frequency of injections, and previous treatment response also play a role in the decision‐making process.

Deciding if and when to stop biologic therapy is controversial. While some children may achieve long‐term asthma control with biologics, there is no evidence how tapering or discontinuing biologics can be done. Premature cessation of biologics could lead to a rebound in exacerbations, necessitating re‐initiation of treatment.^59^

### 3.4 Asthma remission and biologics

The concept of remission refers to the absence of symptoms and exacerbations while maintaining normal lung function. However, current definitions of asthma remission in children are inconsistent and vary widely.^60^ There is ongoing debate whether remission can be defined while on treatment or only after withdrawal, whether lung function and/or biomarkers should be included in the definition, and how long this state must be sustained before it can be formally defined as remission. In adults, “remission” has been considered an achievable goal with long‐term control maintained for more than 1 year. However, in children, the concept of remission is more complex as asthma is a chronic, evolving condition that can fluctuate over time because of immunological maturation, atopy, and environmental exposures.^2^ While control and sustained control are realistic goals in children on biologics, there is debate if complete control on biologic treatment for prolonged periods of time might be called ‘remission’. This underscores the need for standardized definitions of remission and for now a focus on symptom control, QoL, minimizing exacerbations, and stable/highest level lung function rather than the complete absence of disease in the evaluation of treatment effect of biologics. Furthermore, more recent approaches have not only considered remission as absence of disease activity (symptoms, exacerbation, lung function, corticosteroids sparing) but also as absence and/or reversion of chronic inflammation (immune remodeling) and structural damage (bronchial remodeling).^61^ In clinical practice, there are definitely children and adolescents who obtain complete asthma control, are exacerbation‐free with normal lung function and normal biomarkers while on biologics. In which proportion of patients such a complete remission is realistic under biologic treatment and how biologic treatment in that case should be managed may become clearer from real‐world registry studies such as SPACE.^48^

### 3.5 Unmet needs and future research

While biologics are effective for many children with severe asthma, there remain significant unmet needs. First, a substantial proportion of children remains poorly controlled or experiences persistent asthma exacerbations while on biologics,^48^ suggesting that there is a need for new biologics and for guidance on switching biologics. Second, except for Tezepelumab, all biologics target T2 high inflammation, leaving a gap for the (small) group of children with T2 low inflammation. Third, biomarkers that reliably predict treatment response and are able to differentiate best response to a specific biologic are needed. Fourth, there is an unmet need in preschool children with severe wheezing and frequent exacerbations. Dupilumab is currently assessed in a trial in children aged 2 to <6 years (LIBERTY ASTHMA TREKIDS; NCT06191315). The PARK study (Preventing Asthma in high‐Risk Kids) aims to prevent asthma development by early treatment with OMZ in children aged 2–3 years of age with atopy and wheezing (NCT02570984). Last, long‐term safety data is currently lacking. More insight on the adverse effects and influence on the developing immune system in childhood is necessary to evaluate the harms and benefits of biologic treatment in the long term.

New initiatives such as the Severe Pediatric Asthma Collaborative in Europe (SPACE) and PediAsthma in France are providing essential real‐life data on the benefits and safety of biologics in children, also for the long‐term. SPACE is a clinical research collaboration initiated by the European Respiratory Society and includes a registry that explores the use of biologics in children aged 6 to 18 years with SA. It was started on the initiative of the European Medicines Agency (EMA), which observed a lack of participation of children and adolescents with SA in trials of biologics, leading to reduced access to these drugs for children. Cross‐sectional data from SPACE show that most children obtain good symptom control on biologics; still, a substantial proportion of children experienced asthma attacks and hence switching between biologics was common.^48^ The registry presently includes data of more than 500 children on biologics. The PediAsthma study is a large‐scale, 52‐week multicenter real‐world study in France assessing the effectiveness of dupilumab in children aged 6–18 years with severe asthma.^46^ Results from adolescents in this study showed a high burden of disease before start of the biologic and more than half of the patients had been switched from another biologic highlighting an unmet need. Results in school aged children are due in the next few years.

## 4 ATOPIC DERMATITIS

Approximately 10%–30% of children with AD present with moderate‐to‐severe disease. Since 2017, advances in the understanding of T2 inflammation have profoundly transformed therapeutic strategies with the development of biologics and small molecules (Table 2).^10^ In cases of failure of optimized topical therapy (adequate quantity, prolonged duration, early relapse), and after excluding contributing factors such as corticophobia or contact dermatitis, systemic treatment may be considered for moderate‐to‐severe AD, particularly when QoL is significantly impaired.

**TABLE 2:** Main clinical trials and real‐world studies on Systemic Therapies in Pediatric Atopic Dermatitis.

Molecule target	Study (First author, year; and design)	Population	Indication	Treatment (dose, frequency)	Efficacy	Tolerance/Safety
Biologics treatments						
Dupilumab anti–IL‐4Rα	Paller et al.^62^; “LIBERTY AD PED‐OLE”: Open‐label extension	142 children >5 months–6 years	Moderate–severe AD	SC <15 kg: 200 mg Q4W 15–30 kg: 300 mg Q4W	At Week 52: EASI‐75 achieved by 79.3%; IGA 0/1 by 36.2%. Significant improvement in quality of life (CDLQI/IDQOL)	Most common: Nasopharyngitis (19.7%), cough (15.5%), and pyrexia (14.1%). Conjunctivitis in 12.7%. One discontinuation due to severe urticaria
Dupilumab anti–IL‐4Rα	Paller et al.^63^; “PEDIatric STudy in Atopic Dermatitis”: Real‐world cohort	144 children 2–<12 years	Moderate–severe AD	SC 30–60 kg: 200 mg Q2W >60 kg: 300 mg Q4W vs. Methotrexate or ciclosporine	At 2 Years: Dupilumab showed numerically greater EASI improvement (−12.4) vs. Methotrexate (−5.7) or ciclosporine (−3.3). Higher treatment adherence	Dupilumab patients had fewer TEAEs (18.1%) vs. Methotrexate (29.8%) or ciclosporine (31.4%). Conjunctivitis was more common with Dupilumab
Dupilumab anti–IL‐4Rα	Lasek et al.^64^; Retrospective cohort	80 children 6–13 years	Moderate–severe AD	SC weight‐tiered (standard dosing/ATU)	At 12 Weeks: SCORAD 50 achieved by 72.1% and SCORAD 75 by 21.3% of patients. IGA 0/1 reached by 66.7%. Confirms effectiveness in non‐selected patients	Lower frequency of conjunctivitis (11.3%) and facial redness (3.7%) than in adults/adolescents 6.3% discontinued due to eosinophilia or injection pain
Dupilumab anti–IL‐4Rα	Ding et al.^65^; Retrospective cohort	25 children 6–13 years	Moderate–severe AD	SC Weight‐tiered (200 mg or 300 mg) Q4W	At 6 Months: Median EASI improved from 5.9 to 0.7. Median SCORAD from 42.3 to 13.6. Median Pruritus NRS from 7.0 to 2.0. Comorbidities: 80% of asthma patients saw complete disappearance of symptoms.	Adverse effects were mostly mild (e.g., erythema, URTI, conjunctivitis). Therapeutic effect noted as significantly better in children than in adults within the same study.
Tralokinumab anti–IL‐13	Paller et al.^68^; “ECZTRA 6”: phase 3 randomized, double‐blind RCT	298 adolescents 12–17 years	Moderate–severe AD	SC 150 or 300 mg Q2W	At Week 16: EASI‐75: 28.6% (150 mg) and 27.8% (300 mg) vs. 6.4% (placebo). IGA 0/1: 21.4% and 17.5% vs. 4.3% At Week 52: EASI‐75 maintained in >50% of responders. Open‐label phase reached 57.8% EASI‐75	Viral URTI (19.4% with 150 mg), URTI (11.3%), and headache (6.2% Conjunctivitis: Low frequency (~4%) similar to placebo) No treatment‐related serious adverse events
Tralokinumab anti–IL‐13	Villodre Lozano et al.^69^; Spain real‐world cohort	27 adolescents 12–17 years	Moderate–severe AD	SC 300 mg Q2W	At Week 52: Significant EASI reduction (mean 17.7 to 4.6). 83.3% resolution of palmoplantar involvement	Favorable safety profile. Resolution of previous Dupilumab‐induced AEs (erythema, ocular redness) after switching
Lebrikizumab anti–IL‐13	Paller et al.^70^; “Adore”: Open‐label study	206 adolescents 12–17 years	Moderate–severe AD	SC 250 mg Q2W → Q4W after W16	At Week 52: EASI‐75 achieved by 81.9%; IGA 0/1 reached by 62.6%. Improvements in anxiety and depression	Low frequency of SAEs (2.4%) and discontinuations due to AEs (2.4%). Common: Nasopharyngitis and conjunctivitis cluster
Lebrikizumab anti–IL‐13	Hebert et al.^71^; “ADvocate1, ADvocate2, and ADhere.”: Phase 3 RCT subset analysis	99 adolescents 12–17 years	Moderate–severe AD	SC 250 mg Q2W → Q4W after W16	At Week 16: EASI‐75 achieved by 62% (monotherapy) and 88% (+TCS). IGA 0/1 reached by 46.6%	Consistent with adult trials. Conjunctivitis (8.5%) and injection site reactions (2.6%)
Nemolizumab anti–IL‐31Rα	Igarashi et al.^72^; Phase 3 randomized, double‐blind RCT	89 children 6–13 years	Pruritic AD (5‐level itch score >3)	SC 30 mg Q4W (+ topicals)	At Week 16: Significant itch relief from Day 2. Weekly mean 5‐level itch score change: −1.3 vs. −0.5 (placebo)	Manageable safety profile. Most common AEs: worsening of AD (10.9%), upper respiratory inflammation (8.7%), and erythema (6.5%)
Nemolizumab anti–IL‐31Rα	Igarashi et al.^73^; Open‐label extension	89 children 6–12 years	Pruritic AD (5‐level itch score >3)	SC 30 mg Q4W (+ topicals)	At Week 68: Mean EASI change from baseline: −77.1%. Mean change in 5‐level itch score: −1.8.	Safe long‐term use. Frequent AEs: Nasopharyngitis (26%), COVID‐19 (24%), acne (17%), and impetigo (16%)
Small molecules						
Upadacitinib JAK1i	Paller et al.^74^; “Measure Up 1, Measure Up 2, and AD Up”: Analyses of 3 Phase 3 RCTs	542 adolescents 12–17 years	Moderate–severe AD	PO 15/30 mg daily	At Week 76: High efficacy maintained; EASI‐75 achieved by 84%–89% (15 mg) and 82–96% (30 mg) across trials	Frequent acne (8.5–15.1%). CPK elevation (7.1–11.6%). Low rate of herpetic infections (1.1–4.0 events/100PY) No MACE, no VTE No new signals were observed with either dose
Upadacitinib JAK1i	Patruno et al.^75^ Italian real‐world multicenter retrospective	36 adolescents 12–17 years	Moderate–severe AD	PO 15 mg daily	At Week 52: EASI 75/90: 94.1%/77.7%	Acne 15% HSV 6%
Upadacitinib JAK1i	Melgosa Ramos et al.^76^ Spain real‐world multicenter retrospective	21 patients (adolescents+ adults)	Moderate–severe AD	PO	At Week 52: Mean EASI 19.8 → 0.6	No MACE No severe infections
Upadacitinib JAK1i	Zhao et al. 2024^78^ Retrospective cohort	12 children 6–14 years	Refractory AD to Dupilumab	PO 15 mg daily	At Week 16: Superior option for Dupilumab non‐responders; 58.3% achieved EASI‐75	Safety profile favorable; most AEs mild. Common: Acne (33.3%) and infection (25%)
Abrocitinib JAK1i	Eichenfield et al.^79^ “JADE TEEN”: Phase 3 randomized, double‐blind RCT	285 adolescents 12–17 years	Moderate–severe AD	PO 100/200 mg daily	At Week 12: EASI‐75 reached by 68.5% (100 mg) and 72% (200 mg). Fast pruritus relief within 2 days	AE were reported for 59 (62.8%), 54 (56.8%), and 50 (52.1%) patients in the 200 mg, 100 mg, and placebo groups, respectively; nausea was more common with abrocitinib, 200 mg (17 [18.1%]) and 100 mg (7 [7.4%]). Herpes‐related AEs were infrequent; 1 (1.1%), 0, and 2 (2.1%) patients had serious AEs.
Abrocitinib JAK1i	Liang et al.^80^ Chinese prospective real‐world	28 patients 2–17 years	Moderate–severe AD	PO dosing according to the weight 25/50/100 mg daily	At Week 12: EASI‐75 achieved by 60.7%. Significant improvement in itch and sleep	2 adolescents (7.1%) experienced mild adverse events, including Kaposi's varicelliform eruption and nausea, with no occurrence of serious adverse events throughout the treatment period
Abrocitinib JAK1i	Paller et al.^81^ “JADE MONO‐1, MONO‐ 2, TEEN, REGIMEN +phase 3 extension trial, EXTEND”	357 patients 2–17 years	Moderate–severe AD	PO 100/200 mg daily	At Week 112: Sustained efficacy; ~85% achieve EASI‐75. Comparable responses between doses	Consistent long‐term safety profile. Serious TEAE (100/200): 7%/10.4% Serious infections: 2%/3.5% Herpes zoster infection: 3%/4.2% Acne 7.6% both group Nausea (100/200): 8%/18.7% VTE (1) (200 mg) MACE (1) (100 mg)
Baricitinib JAK1/2i	Wollenberg et al.^82^ “BREEZE‐AD‐PEDS” Phase 3 extension trial	476 children 2–17 years	Moderate–severe AD	PO 1/2/4 mg daily	At Week 52: Sustained long‐term efficacy; IGA 0/1 achieved by 56.8% and EASI 75 by 71.6% (4 mg equivalent)	TEAE 77.7% (36% mild 35.5% moderate) Upper respiratory tract infections 9.2% Herpes zoster 1.5% Herpes simplex 6% No mace no thrombosis no cancer
Ivarmacitinib JAK1i	Zhao et al.^83^ Phase 3 randomized, double‐blind RCT	336 patients ≥12 years (adolescents and adults)	Moderate–severe AD	PO 4/8 mg daily	At Week 16: EASI‐75 achieved by 54% (4 mg) and 66.1% (8 mg). Fast Wi‐NRS response by Week 1	TEAT 69.0% and 66.1% (for 4 and 8 mg). URTI (11%), CPK elevation (7%–10%), and acne (3.5%–6.3%). No MACE no thrombosis

Abbreviations: AD, atopic dermatitis; EASI, Eczema Area and Severity Index; SC, sub cutaneous; PO, per os; W, week; Y, year; M, month; RCT, randomized control trial; IGA, investigator global assessment; TEAE, treatment emergent adverse event; AE, adverse event; URTI, Upper respiratory tract infection; VTE, venous thromboembolism event; MACE, major adverse cardiovascular event; JAKi, janus kinase inhibitors; QW, quaque week; WP‐NRS, Worst Pruritus Numerical Rating Scale; anti IL4R/3/31 RA, anti interleukin 4 receptor/3/31 receptor alpha; TCS, topical corticosteroid.

### 4.1 Biologicals

Dupilumab was the first biologic approved for pediatric AD. Real‐world studies and open‐label extension trials have confirmed its efficacy and safety as a first‐line systemic treatment in children older than 6 months.^62^, ^63^, ^64^, ^65^ Adverse events include injection‐site reactions, head and neck dermatitis, and conjunctivitis (approximately 10%), without an increased risk of serious infections.^62^, ^63^, ^64^ Because dupilumab is also approved for SA in patients older than 6 years, it improves overall disease control in children suffering from both conditions.^40^, ^66^, ^67^

Tralokinumab and lebrikizumab, both anti–IL‐13 monoclonal antibodies, have demonstrated efficacy in adolescents older than 12 years, with lower rates of conjunctivitis compared with dupilumab.^68^, ^69^, ^70^, ^71^ Long‐term studies and head‐to‐head real‐life comparisons may clarify the best indications according to phenotype, age, and safety (ocular diseases, arthralgia, head and neck involvement, and infections)^69^ between anti–IL‐13 and dual IL‐4/IL‐13 blockade. Nemolizumab, an anti–IL‐31Rα monoclonal antibody, is approved for patients older than 12 years and has shown a favorable safety profile (conjunctivitis 0.6%, herpes infections 1.1%). By targeting the pruritus pathway, it improves itching, sleep disturbance, and QoL, including in children older than 6 years, although its effect on inflammatory skin lesions appears slower.^72^, ^73^ Emerging therapies targeting the OX40/OX40L co‐stimulatory pathway are currently under investigation in pediatric AD.

### 4.2 Small molecules

JAK inhibitors, including upadacitinib,^74^, ^75^, ^76^, ^77^, ^78^ abrocitinib,^77^, ^79^, ^80^, ^81^ baricitinib,^82^ and ivarmacitinib^83^ have demonstrated rapid efficacy in adolescents (≥12 years) and, for baricitinib, in younger children (>2 years). Reported adverse events mainly include viral infections (Table 2). While major adverse cardiovascular events, venous thromboembolism, and malignancies have been reported in adults, these events remain rare in pediatric populations to date. Nevertheless, appropriate pre‐treatment assessment and monitoring are required, including evaluation of infection risk and live vaccine contraindications.

### 4.3 Choosing the appropriate treatment strategy

Meta‐analyses in children aged 2–18 years show broadly comparable efficacy between biologics and JAK inhibitors, with slight superiority reported for dupilumab, lebrikizumab, and upadacitinib in some analyses,^84^, ^85^, ^86^, ^87^ including a dose–response relationship for upadacitinib.^88^ However, given the broader immunomodulatory effect and the increased infection risk observed in adults receiving JAK inhibitors, biologics are often preferred for long‐term pediatric management. Thus, biologics and JAK inhibitors should be considered complementary strategies: JAK inhibitors for rapid symptom control; biologics for sustained disease control; multimorbidity management (e.g., dupilumab), and therapeutic repositioning (switching) in cases of loss of response or adverse events (e.g., head and neck dermatitis or conjunctivitis under dupilumab) (Table 3).

**TABLE 3:** Comparison between biologics and JAK inhibitors in pediatric atopic dermatitis.

Treatment	Biologics	Small molecules (JAK inhibitors)
Pre‐treatment assessment	Check vaccination status (live attenuated vaccines) None mandatory (minimal laboratory tests if clinically indicated)	Check vaccination status CBC, renal, hepatic and lipid panel; viral serologies (HBV, HCV, HIV) Consider TB screening Contraception counseling in adolescents
Route of administration	Subcutaneous injection	Oral
Dosing frequency	Every 2–4 weeks (depending on molecule and weight)	Once daily
Targeted pathways	Targeted Th2 blockade (IL‐4Rα, IL‐13, IL‐31Rα)	JAK–STAT pathway inhibition
Common adverse events	Injection‐site reactions; conjunctivitis; head and neck dermatitis; mild eosinophilia; rare herpes infections	Acne; viral infections (HSV, herpes zoster); laboratory abnormalities (lipids, CPK); increased risk of MACE/VTE and cancer in elderly patients with comorbidities, but pediatric studies are reassuring.
Laboratory monitoring	No routine laboratory monitoring required	Regular monitoring of CBC, liver enzymes, and lipid profile
Main advantages	Long‐term safety data; sustained disease control; effective in T2 comorbidities; lower systemic immunosuppression	Rapid onset of action (especially on pruritus); strong short‐term efficacy; oral administration; useful in difficult phenotypes
Limitations	Injection route; Ocular adverse events in some patients	Increased infection risk; need for laboratory monitoring; rebound after discontinuation

Abbreviations: CBC, complete blod count; CPK creatine phosphokinase; HBV, hepatitis B virus; HCV, hepatitis C virus; HIV, human inmmunodeficiency virus;MACE/VTE, major adverse cardiovascular event/venous thromboembolism; JAK‐STAT, Janus kinase, signal transducerand activator of transcription protein; TB : tuberculosis.

### 4.4 Discontinuation

According to the immune target, relapse after treatment withdrawal differs. In a real‐world cohort, the median time to relapse was 60 days with JAK inhibitors versus 457 days with dupilumab, with relapse rates of 52.9% and 34.6%, respectively.^89^ These data suggest that JAK inhibitors suppress inflammation during exposure, whereas IL‐4/IL‐13 blockade provides more sustained disease control. Consequently, while discontinuation of JAK inhibitors is followed by early rebound, spacing strategies with biologics may be feasible. A French randomized trial is underway to analyze spacing of dupilumab doses (NCT05642208).

### 4.5 Dupilumab and risk of type 2 comorbidities

Analyses of clinical trial datasets show reduced acquisition or worsening of type 2 inflammatory diseases and IgE sensitization, while pediatric real‐world cohorts report a lower 3‐year incidence of asthma and allergic rhinitis compared with conventional therapies. Definitive prevention remains unproven.^90^, ^91^, ^92^

## 5 CHRONIC URTICARIA

Pediatric CSU endotypes are poorly studied and are currently divided into autoallergic, autoimmune, and idiopathic subtypes. OMZ, an anti‐IgE monoclonal antibody, is an effective treatment for antihistamine‐refractory pediatric CSU and has marketing authorization for patients >12 years old. In practice, real‐world pediatric use extends to children aged 5–11 years, even though randomized controlled studies assessing its use at this age are lacking.^93^ A meta‐analysis including 36 studies reported an overall response rate of 88.0% with OMZ, a complete response rate of 51.0%, and a relapse rate of 24.3%, with few adverse events (3.4%).^94^ New therapeutics for non‐responders are emerging: dupilumab has recently been approved by the FDA for adolescents and adults, and BTK inhibitors, such as remibrutinib, have shown promising results in adults.^95^ Early onset of CSU, longer pre‐treatment symptom duration, and longer disease course may predict persistence, supporting early, targeted, and effective intervention.^96^

## 6 FOOD ALLERGY

**FIGURE 3:**
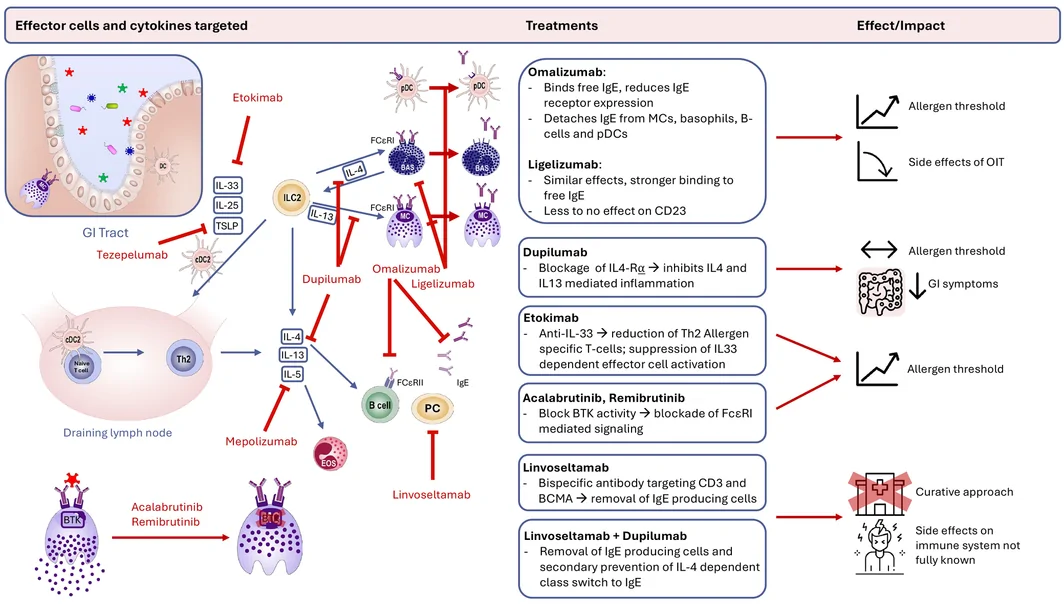
Biologics and small molecules effects in food allergy. BAS, basophil; BCMA, B cell maturation antigen; BTK, Bruton tyrosine kinase; cDC, conventional dendritic cell; MC, mast cell; PC, IgE producing plasma cell; ILC2, group 2 innate lymphoid cell.

Biologics are used either as monotherapy or in combination with allergen‐specific oral immunotherapy (Figure 3). No approach has provided evidence of long‐lasting disease modification once the drug is no longer administered. Combination with allergen‐specific immunotherapy carries the downside of a double treatment burden but also the advantage of being very specific and the ability to create sustained effects in a sub‐population of patients with FA.

### 6.1 Anti‐IgE biologics

#### 6.1.1 Omalizumab monotherapy

In FA, OMZ monotherapy aims to increase the amount of food tolerated without allergic symptoms, thereby reducing accidental allergic reactions, improving QoL and limiting anxiety, social isolation, and lifestyle restrictions. Studies evaluating the efficacy and safety of OMZ monotherapy in FA are listed in Table 4.^11^, ^12^, ^97^, ^98^, ^99^, ^100^, ^101^, ^102^, ^103^, ^104^, ^105^, ^106^ Most of the participants were children or adolescents.

**TABLE 4:** Studies on anti‐IgE in food allergy.

Ref	Type of study	Number of patients	Age	Intervention	Dosage	Duration	Food	Reaction threshold	Timing of post‐treatment OFC	Endpoints	Results	Safety
Rafi et al.^98^	Prospective pilot study	*N* = 22	14–66 years (*n* = 5 < 18)	OMZ therapy for food allergy and asthma	150–300 mg every 2–4 weeks Based on weight and total IgE	At least 1 year	Fish, shellfish, peanut, tree nuts, egg, soybean, wheat		No data	Primary: Reduction or elimination of allergic symptoms upon accidental or intentional re‐exposure to sensitized foods. Secondary: Symptom‐specific improvements and safety	Primary: All patients reported a decrease or absence of symptoms upon accidental or intentional re‐exposure Secondary: 100% of patients (22/22) showed improvement by the 6th dose.	
Sampson et al.^99^	Phase II, randomized, double‐blind, parallel‐group, placebo‐controlled oral food challenge trial	*N* = 14 Early termination (150 planed)	6–75 years (*n* = 7 6–12 years)	OMZ vs. Placebo	Minimum of 0.016 mg/kg/IgE every 4 weeks OMZ < =300 mg Every 2 weaks OMZ > 300 mg	24 weaks	Peanut flour	<250 mg of peanut flour at baseline	After 24 wks	Primary: tolerance of ≥1000 mg of peanut flour after 24 weeks Secondary: improvement in the tolerated dose	Primary: 44.4% of OMZ‐treated subjects vs. 20% in placebo. Secondary: 80.9‐fold improvement in the tolerated dose in OMZ group vs. a 4.1‐fold improvement in the placebo group	Good safety profile for OMZ but severe reaction during DBPCFC
Savage et al.^100^	Open label study, phase 2	*N* = 14	Median age, 23 years (18‐50 years)	OMZ monotherapy for 6 months in peanut allergic patients	According to standard asthma dosing	6 months	Peanut	<1000 mg of peanut protein		Primary: increase tolerance of peanut protein after 6 months of treatment	Primary: median cumulative eliciting dose of peanut protein significantly increased from 80 mg (range, 30–380 mg) to 6500 mg (range, 1830–10,000 mg)	No data
Brandström et al.^101^	Open‐label, phase 2 study First part (OMZ treatment)	*N* = 23	12–19 years	OMZ Individually dosed based on basophil activation (CD‐Sens)	Initially 450–675 mg every 4 weeks Up to 600 mg every 2 weeks	8–24 weeks, depending on CD‐sens values	Peanut	Positive OFC to less than 10,000 mg (*n* = 13, positive OFC from 28 to 840 mg of peanut protein) or convincing history of anaphylaxis in the past 5 years (*n* = 10)	Negative or favorable CD‐Sens test	Primary: the ability to undergo an open peanut challenge without severe allergic reactions. Secondary: The peanut protein dose tolerated during the challenge	Primary: all patients continued with an open peanut challenge with no (*n* = 18) or mild (*n* = 5) objective allergic symptoms Secondary: Peanut challenge tolerance increased 50‐fold on average All patients tolerated >840 mg of peanut protein	No data
Fiocchi et al.^12^	Single‐center, single arm, real‐life, retrospective observational study	*N* = 15 pediatric patients with severe asthma	8–23 years	OMZ monotherapy for 4 months	According to standard asthma dosing	16–24 weeks	37 foods (milk, egg, wheat, hazelnut, …)	Median (IQR) eliciting threshold 460.8 mg of protein (31–1740)	After 4–6 months of OMZ	Primary: increase in food allergen thresholds Secondary: immediate reactions to 2 foods or more before and after a 4‐month treatment with omalizumab.	Primary: increase in the allergen threshold for milk, egg, wheat, and hazelnut from a mean 1012.6 ± 1464.5 mg protein to 8727 ± 6463.3 eliciting dose (*p* < .001). Secondary: 70.6% of subjects tolerated the complete challenge dose (serving size) and foods were reintroduced without OIT.	
Crespo et al.^102^	Observational study	*N* = 5	4–8 years	OMZ in children with multiple FA	150–300 mg every 4 weeks; adjusted per patient	2.3–6.2 years	Various foods: nuts, fish, vegetables, fruits, and cereals			Primary: tolerance to previously allergenic foods Secondary: Introduction of foods previously excluded due to anaphylaxis.	Primary: Significant increase in food threshold Secondary: reduction in allergic reactions, improved quality of life	
Chinuki et al.^103^	Phase 2, multicentre single‐arm trial	*N* = 20	Adult patients	OMZ	According to standard asthma dosing	12 months	Wheat‐dependent exercise‐induced anaphylaxis (WDEIA)	No OFC	No OFC, consumption of products after lifting of restrictions on wheat intake	Primary: % of patients achieving a BAT rate below 10% Secondary: % of patients with an allergic score of 0 when wheat products were consumed after lifting of restrictions on wheat intake (when BAT level <10%)	Primary: 8/11 patients with hydrolyzed wheat protein‐WDEIA (73%) and 8/8 patients with conventional‐WDEIA (100%), A BAT suppression over 70% was achieved for both groups Secondary: % of patients with an allergic score of 0 when wheat products were consumed: 6/8 patients with hydrolyzed wheat protein‐WDEIA (75%) and 5/8 with conventional‐WDEIA (63%)	During OMZ, allergic symptoms due to wheat ingestion in 5/16 cases (average allergy score of 1.6, 1 patient with score 3). After OMZ cessation, allergic symptoms due to wheat ingestion in 13 cases (average allergy score of 1.4, 1 patient with score 4). No severe AE
Dinardo et al.^104^	Case series	*N* = 7	7–18 years	OMZ for severe asthma with an IgE level of >1500 and food allergies	Dosage adjusted per baseline total IgE and weight	≥2 years	Cow's milk, egg, peanuts	Initial mean threshold: 21.72 ± 11.92 mg of food protein	1 and 2 years after OMZ	Secondary: increase in reactivity mean threshold at 1 and 2 years of treatment	Reactivity mean threshold increased up to 355 ± 3195 mg at 1 year, and 4505 ± 3117 mg of food protein at 2 years	Headache and fever 24 h after the ad‐ministration in one patient, this patient continued the treatment withoutany further adverse event.
Mortz et al.^105^	DBRPC^a^	*N* = 20	6–17 years	OMZ monotherapy	Asthma‐based dosing	3–6 months	One FA (Peanut, hazelnut, cashew, walnut, egg)	13–443 mg food protein		Primary: increase in threshold dose after 3 months of treatment	All treated participants increased tolerance levels ≥2 challenge steps (*p* = .003). At 6 months, many children tolerated very high doses (up to 44,000 mg of food protein).	
Wood et al.^11^	DBRPC	*N* = 180	1–55 years *N* = 177 1–17 years	OMZ monotherapy	300 mg every 2 wks or 225 mg every 4 weaks based on weight and total IgE levels	16 to 20 weeks; additional 20–24 weeks	Multiple (Peanut, cashew, milk, egg, walnut, wheat, hazelnut)	<100 mg of singledose peanut protein and <300 mg of single‐dose food protein for other allergens	16–20 weaks after OMZ initiation	Primary: tolerance of at least 600 mg of peanut protein in a single dose after 16–20 weeks of treatment. Secondary: Tolerance of at least 1000 mg of cashew, milk, and egg (each); tolerance to increasing cumulative doses of one, two, or all three foods, up to a total of 6044 mg	Primary: 67% achieved tolerance vs. 7% placebo. Secondary: tolerance to a single dose of ≥1000 mg of food protein was achieved in the OMZ group, by 41% for cashew (28/68) vs. 3% (1/31), 67% for egg (34/51) vs. 0% (0/20), and 66% for milk (27/41) vs. 10% (2/21). No significant change in QoL	
Arasi et al.^106^	Observational study	*N* = 69	6–18 years	OMZ therapy for food allergy and asthma	Based on weight and total IgE	1 year	One FA or multiple FA: Peanut, tree nuts, fish, egg, milk, wheat		4 months	Primary: Increase in threshold of reactivity (NOAEL) to food allergens after 4 months of OMZ treatment (T1) Secondary: NOAEL increase at T2 and T3 in patients who failed T1 challenges.	Primary: complete clinical desensitization to 66.4% of tested foods after 4 months of treatment Secondary: a further increase to 22.4% by 1 year, reaching a total of 88.7%.	

Abbreviations: AE, Adverse event; BAT, Basophil activation test; DBRPCT, double‐blind randomized placebo‐controlled trial; FA, Food allergy; IgE, IL, Interleukin; Immunoglobulin E; IQR, Interquartile range; OFC, Oral food challenge; OMZ, Omalizumab; QoL, Quality of Life; NOAEL, No‐observed‐adverse‐effect level; OIT, Oral immunotherapy; WDEIA, wheat‐dependent exercise‐induced anaphylaxis.

The OUTMATCH trial is a phase 3, randomized, double‐blind, placebo‐controlled (DBPC) study evaluating OMZ monotherapy using an asthma dosing table. At week 16, 67% of OMZ‐treated patients tolerated 600 mg peanut protein versus 7% receiving placebo (*p* < .001). Similar results were observed for other foods (milk 66% vs. 10%; egg 67% vs. 0%; *p* < .001), less for cashew (41% vs. 3%).^11^ Real‐life and meta‐analyses studies confirmed these findings. In children with severe asthma and multiple food allergies, OMZ increased thresholds of reactivity and reduced accidental reactions.^12^, ^102^ A first meta‐analysis (4 OMZ monotherapy studies) reported a significant increase in the tolerated dose of multiple foods after 17–22 weeks OMZ treatment [Risk ratio (RR): 24.88; *p* < .001].^107^ Another highlighted that OMZ significantly improved desensitization rates and increased reactivity thresholds compared to placebo (RR: 2.035 and RR 4.90, respectively), reducing the risk following food exposure (RR: 0.55).^108^

Regarding QoL, there was no significant improvement (FAQLQ score) in the 16 weeks blinded phase of OUTMATCH trial, but there were some positive effects in the 24 weeks open‐label extension.^11^ In the real life study by Fiocchi et al., the QoL (PedsQL questionnaire) significantly increased, reflecting a better asthma control and a reduction in dietary restrictions.^12^ This was also clearly observed in another open‐label OMZ trial in asthmatics with FA (FAQLQ questionnaire).^106^

OMZ using a dedicated dosing table was approved by the FDA in February 2024 to reduce allergic reactions (including anaphylaxis) following accidental exposure to one or more foods in adults and children aged ≥1 year with IgE‐mediated FA, in combination with continuation of food avoidance. OMZ is also now incorporated in the EAACI's FA treatment guidelines.^109^, ^110^ Despite these advances, none of the OMZ trials demonstrated a clear disease modifier effect. Consequently, research focused on combined treatment with oral immunotherapy (OIT) to improve efficacy and long‐lasting benefit.

#### 6.1.2 Omalizumab treatment in combination with OIT

OMZ was investigated in combination with OIT to increase allergen threshold before OIT initiation, to reduce OIT adverse events, and to improve efficacy and safety of mono or multi‐food OIT. The ultimate goal is to decrease the time to reach the maintenance dose or the complete desensitization. Several studies have shown these effects compared to OIT without OMZ (Table 5).^111^, ^112^, ^113^, ^114^, ^115^, ^116^, ^117^, ^118^, ^119^, ^120^, ^121^, ^122^, ^123^ Importantly, up to now, there remains a lack of evidence that OMZ enhanced the effect of OIT on tolerance development.

**TABLE 5:** Studies on anti‐IgE with OIT in food allergy.

Ref	Type of study	Patients number and age	Intervention	Dosage	Duration	Food	Reaction threshold	OIT	Timing of post‐treatment OFC	Endpoints	Results	Safety
Nadeau et al.^111^	Pilot phase I study	*N* = 11 children. 7–17 years, median age: 8 years	OMZ + OIT	Based on weight and IgE level for children with IgE levels <700 kU/L, 0.016 mg/kg/IgE [U/mL] for the 3 children with serum IgE levels >700 kU/L, every 2–4 weeks	16 weeks	CM	No OFC at inclusion cow's milk–specific IgE >25 kUA/L, total IgE <2500 kU/L; with significant clinical history of IgE mediated cow's milk allergy	Week 9: rush oral desensitization followed by desensitization with daily doses of milk continued in 10/11 subjects, with weekly increases in the dose of milk over the next 7–11 weeks	Week 24	DBPCFC: 5 doses, up to 3000 mg (cumulative dose, 7250 mg, equivalent to 220 mL milk).	9/11 patients reached a daily dose of 2000 mg All 9/11 passed the DBPCFC and an open challenge All 9/11 patients continued with daily milk ingestion >8000 mg/d, which included different types of milk products	Mean frequency for total reactions by week 24: 1.6% (32 reactions of 2199 doses total for all 11 subjects). All patients experienced some adverse events, mild (1%), moderate (0.3%), and severe (0.1%) 1% local reactions (mostly pruritus or urticaria) and/or gastrointestinal (e.g., abdominal pain)
Schneider et al.^4^	Open‐label study	*N* = 13 children 8‐16y (median age: 10 years), *N* = 6 with current eczema, asthma, or both, *N* = 6 with at least one other FA	OMZ + OIT	Weight and total IgE level within the dosing guidelines for OMZ	20 weeks of OMZ 20 weeks of OIT (started at week 12)	Peanut	DBPCOFC: ≤100 mg peanut flour (cumulative dose of ≤186 mg)	Week 12: rush desensitization day (cumulative dose 992 mg peanut flour). Next day: 500 mg, followed by weekly increase from 500 mg to 4000 mg peanut flour daily	Week 32	Primary: Final DBPCFC: tolerance of cumulative dose of 8000 mg peanut flour.	All 13 subjects tolerated the rush desensitization 12 subjects reached the maximum maintenance dose of 4000 mg peanut flour per day in a median time of 8 weeks All 12 subjects tolerated a challenge with 8000 mg peanut flour (160 to 400 times the dose tolerated before desensitization).	Mild or no allergic reaction in 6/13: Grade 3 reactions in 2/13 subjects rapid response to treatment
Yee et al.^113^	Long‐term follow‐up study				Follow‐up for 72 months (67 months of maintenance)			Daily maintenance dose (varied between 500 and 3500 mg, different peanut‐containing products)		OIT continuation, adverse events, biomarkers change, QoL	7/13 patients continued on peanut OIT through month 72; 6/13 discontinued therapy because of adverse reactions (they had higher peanut‐IgE and Arah2‐IgE at study month 12). Patient and parent QoL improved from baseline, even in patients who discontinued OIT.	OIT tolerance: All patients experienced at least 1 adverse event, 1 patient developed EOE
Bégin et al.^114^	Open‐label, phase 1 study	*N* = 25 participants 4 years and older (median age 7 years)	OMZ + OIT	Based on weight and total IgE levels as per Omalizumab Global Dosing schedule	16 weeks	Multiple: cow's milk, egg, peanut, nuts, grains and sesame seed	Positive allergic reaction in a DBPCFC at a protein dose <100 mg	Week 8: rush OIT to up to 5 allergens simultaneously	No OFC	Safety Dose tolerability	19 participants tolerated all 6 steps of the initial escalation day (up to 1250 mg of combined food proteins), requiring minimal or no rescue therapy. 6 participants started on their highest tolerated dose as their initial daily home doses. Maintenance dose of 4000 mg protein per allergen reached at a median of 18 weeks	401 reactions per 7530 home doses (5.3%) median of 3.2 reactions per 100 doses (mild: 94%, 1 severe)
Wood et al.^115^	DBPCT	*N* = 57 subjects 7–35 years of age, 3 sites	OMZ vs. Placebo (randomization 1:1)	Dose based on the dosing chart, or on 0.016 mg/kg/IgE IU, every 2–4 weeks	24 months (12 months for Placebo, stopped after unblinding)	Cow's milk	DBPCFC: reaction to <2000 mg of milk protein, median successfully consumed dose 20 mg in the OMZ group, 10 mg in the placebo group	Month 4 to month 26: open‐label milk OIT dosing administered using non‐fat dry powdered milk, Daily home dosing, escalated every 2 weeks for 22–40 weeks, up to a minimum maintenance dose of 520 mg milk protein (15 mL of liquid milk).	Month 28 (desensitization OFC), Month 30 (discontinued milk OFC), Month 32 (tolerance OFC): 10 g DBPCFC	Primary: Sustained unresponsiveness (SU): absence of dose‐limiting symptoms in both the month‐28 and −32 OFCs (after 8 weeks off of milk OIT) Secondary: successfull desensitization (absence of dose‐limiting symptoms in the month‐28 OFC), change in OFC successfully consumed dose (SCD), time and number of doses to achieve maintenance, changes in allergic tests	Primary endpoint: At month‐28, 24 (88.9%) omalizumab‐treated subjects and 20 (71.4%) placebo‐treated subjects passed the 10 g “desensitization” OFC (*p* = .18). At month‐32, SU in 48.1% in the OMZ group vs. 35.7% in the placebo group (*p* = .42). Secondary endpoints: escalation in OMZ subjects for percent doses/subject provoking symptoms (2.1% vs. 16.1%; *p* = .0005), dose‐related reactions requiring treatment (0.0% vs. 3.8%, *p* = .0008), and doses required to achieve maintenance (198 vs. 225; *p* = .008)	Overall percent of symptom‐free doses during escalation: 91.5% among OMZ subjects vs. 73.9% for placebo subjects (*p* < .0001); Adverse reactions markedly reduced during OIT escalation in OMZ subjects for percent doses/subject provoking symptoms (2.1% versus 16.1%; *p* = .0005)
MacGinnitie et al.^116^	Phase II study, multi‐center	*N* = 37 (median age: 10 years) omalizumab (*N* = 29) placebo (*N* = 8)	OMZ + OIT Randomization: 3.5–1 ratio	Based on weight and IgE level (dose table slightly modified from that used for asthma)	20 weeks	Peanut	DBPCOFC: ≤50 mg peanut protein dose (cumulative dose of 88 mg); median cumulative eliciting dose: 38 mg (0.5–88 mg) in the OMZ group and 88 mg (13–88 mg) in the Placebo group	Week 12: rush desensitization day (cumulative dose 490.5 mg peanut floor). Next day: highest tolerated dose once daily home for the next week. Weekly up‐dosing; up to 2000 mg of peanut protein.	Week 31	Final DBPCFC: 3 doses, cumulative dose 4000 mg peanut flour.	Median peanut dose tolerated on the initial desensitization day: 250 mg for omalizumab versus 22.5 mg for placebo treated subject. 23/29 (79%) subjects on OMZ tolerated 2000 mg peanut protein 6 weeks after stopping OMZ versus 1/8 (12%) on placebo (*p* < .01). 23 subjects on OMZ versus 1 on placebo passed the 4000 mg food challenge.	Reaction rates were comparable between OMZ and placebo groups (OR = 0.57 *p* = .15), although OMZ treated subjects were exposed to much higher doses of peanut. *N* = 3 subjects who stopped peanut dosing because of persistent GI symptoms (2 EOE confirmed by biopsy)
Takahashi et al.^117^	Prospective RCT	*N* = 16 (6–14 years)	OMZ + OIT Randomization: 1:1 vs. untreated	Chart‐based dose, every 2–4 weeks Or 1500 IU/mL/body weight if total IgE >1500 IU/mL	24 weeks	Cow's milk	Positive DBPCFC to CM: Sampson's symptoms score > grade 2 with below 10 mL, (4) sIgE level to CM >17.5 kUA/L	Week 8: escalation of OIT followed by maintenance phase until week 32	Week 32	Final DBPCFC: total dose of 200 mL	None of the 6 children in the untreated group developed desensitization to CM while all of the 10 children in the OIT‐OMZ treated group achieved desensitization (*p* < .001). A significantly decreased wheal diameter in response to a skin prick test using CM was found in the OMZ‐OIT treated group (*p* < .05).	Escalation phase of OIT (at hospital): 0.012 adverse reactions per dose per child At home, during the following year: 0.048 adverse events per dose per child No anaphylactic shock or death
Andorf et al.^118^	Blinded, phase 2 clinical trial	4–15 years *N* = 48 randomized 12 non‐randomized, untreated controls	Randomization (3:1) *n* = 36: multifood OIT to 2–5 foods + OMZ *n* = 12: multifood OIT to 2–5 foods + Placebo	According to manufacturer's instructions and the product insert	16 weeks	Multiple: cashew, walnut, hazelnut, almond, sesame, cow's milk, hen's egg, peanut, soy, and/or wheat	Positive DBPCFC to the offending food	Week 8: multi‐OIT to a minimum of 2 and a maximum of 5 allergens simultaneously	Exit DBPCOFC at week 36	Primary: % of participants who passed DBPCOFC to ≥2 of their offending foods	Primary endpoint: 30/36 (83%) in the OMZ group vs. 4/12 (33%) in the placebo group passed DBPCFCs to 2 g protein for ≥2 of their offending foods (OR: 10, 95% [1.8, 58.3], *p* = .004). The same individuals also tolerated 4 g protein of ≥2 foods (*p* = .004).	No serious AEs nor any severe AEs
Andorf^119^	Multicenter randomized, DBPC phase 2 trial (36 weeks)	*N* = 70 5–22 years (mean age: 9.8 years)	OMZ + multiOIT Phase 1: open‐label phase OMZ week 0–16 OIT week 8–29 Phase 2 Randomization 1:1:1 to continue OIT 1 g, 300 mg, 0 mg arms week30‐36	Chart‐based dose, every 2 to 4 weeks	16 weeks	Multiple: cashew, walnut, hazelnut, almond, sesame, cow's milk, hen's egg, peanut, soy, and/or wheat	Positive DBPCFC to the offending food At ≤125 mg dose, equivalent to a cumulative total of 500 mg food protein.	Week 8: multi‐OIT to a minimum of 2 and a maximum of 5 allergens simultaneously to reach a maintenance dose of ≥1 g of each allergen Week 30: Blinded randomization 1:1:1 to continue OIT 1 g, 300 mg, 0 mg (inactive placebo made up of oat flour) arms week 30–36	Week 30 and week 36 OFC	Primary: % of participants who tolerated at least 2 g protein to at least 2 food allergens at week 36. Sustained desensitization to 2 g protein to at least 2 food allergens Secondary: % of participants who tolerated at least 2 g protein to their 3, 4, 5 food allergens at week 36. % of participants who passed the OFC at week 36 (4 g protein of each of 2 food allergens).	Primary: Most participants were able to reach a dose of ≥2 g of each of 2, 3, 4, and 5 food allergens at week 36 Secondary: No difference between a 300 mg dose and a 1 g dose in maintaining desensitization, both together more effective than OIT discontinuation (0 mg dose) (85% vs 55%, *p* = .03). 55%/69% of participants (ITT/per‐protocol) randomized to the 0 mg arm showed no objective reactivity after 6 weeks of discontinuation. Cross‐desensitization between cashew/pistachio and walnut/pecan when only one of the foods was part of OIT.	No clinically significant differences in AE rates between the 1 g, 300 mg, 0 mg discontinuation and non‐randomized arms. Antihistamine and inhaler use were similar across arm. Higher number of doses of injectable epinephrine occurred in the 1 g (4 doses) vs discontinuation (1 dose) vs 300 mg (no doses) arms. No cases of life‐threatening anaphylaxis or eosinophilic esophagitis.
Brandström al.^120^	Open‐label, phase 2 study Second part	*N* = 23 (12–19 years)	OMZ + peanut OIT	Individually dosed OMZ based on CD‐sens monitoring. Initially 450–675 mg every 4 weeks Up to 600 mg every 2 weeks	8–24 weeks, according to CD‐sens test	Peanut	Positive OFC to less than 10,000 mg (*n* = 13, positive OFC from 28 mg to 840 mg of peanut protein) or convincing history of anaphylaxis in the past 5 years (*n* = 10)	Peanut OIT increased from 280 to 2800 mg peanut protein in 8 weeks followed by an individualized step‐wise withdrawal of OMZ, based on clinical symptoms and CD‐sens levels.	20 weeks of pOIT (12 weeks without OMZ)	Primary: daily dose of 2800 mg of peanut protein 12 weeks after the discontinuation of OMZ Secondary: proportion of subjects with a reduced pOIT dose, proportion of subjects not able to discontinue OMZ	Primary: 9/23 (39%) were still able to consume the daily dose of 2800 mg 12 weeks after discontinuation of OMZ. All 23 subjects reached the 2800 mg maintenance dose in a median time of 10 weeks Secondary: 2/23 completed the study with a reduced pOIT dose due to taste aversion 6/23 not able to discontinue OMZ 6/23 withdrew from the study (1EoE, 2 lack of motivation and aversion, 3 fear of reaction and aversion).	Systemic reactions due to peanut doses in 12/17 patients (70.6%) including one patient with grade 3‐ anaphylaxis (after discontinuation of OMZ). In patients who were able to discontinue OMZ, most of systemic reactions (22/27) occurred when OMZ had been decreased by more than 75%.
Ibáñez‐Sandín et al.^121^	OmaBASE: national, multicenter, real‐life open label, and observational registry	*N* = 51 Median age: 10.3 years (IQR, 6.3–13.2 years)	OMZ + CM OIT	According to the investigator's criteria. Median dose 300 mg/months (range, 75–1200 mg/months).	Median OMZ duration: 7.5 months (IQR, 5.0–13.0). OMZ continued in 16 (42%): 8 with reduced dose, 8 maintaining the same dose	Cow's milk	Patients of any age allergic to CM: OFC in 23 (45%)	After a median of 4 (IQR, 2–7) months of OMZ treatment, OIT up‐dosing (UP): dose increments every 1–2 weeks up at hospital to a minimum dose of 6000 mg of CMP, Followed by OIT maintenance phase (MP) (at least 6000 mg of CMP)	No systematic post treatment OFC Median of 16 months (IQR, 12–30) of follow‐up without OMZ	Effectiveness and AE of multiOIT under OMZ and after omalizumab dose modification in real life	After a median of 4 (IQR, 2–7) months of OMZ treatment, 43 patients started UP MOIT. All patients (43/43) reached the target programmed dose (≥6000 mg of CMP) at the end of UP. At database closure, 17 of 42 patients (41%) had discontinued OMZ: 14 patients (33%) could tolerate ≥6000 mg CMP (≥200 mL), and 3 patients (7.2%) 1500–4500 mg CMP (50–150 mL), 21 (50%) were still receiving OMZ: 20 (48%) tolerated ≥6000 mg CMP and 1 (2%) 1500–4500 mg CMP.	AE by CMP doses in 51% rhinitis as the most frequent symptom 2 epinephrine shots AE in 59% of patients that discontinued OMZ and in 25% of those who did not stop it (*p* = .013). Anaphylaxis in 36% of patients after OMZ discontinuation
Sindher et al.^122^	Phase 2, randomized DBC multi‐OIT with OMZ‘real life’ study (2 centers)	*N* = 60 participants aged 2–25 years (median age: 10 years)	OMZ + multi‐OIT Week −8 – 0: OMZ phase (open‐label) Week 0: Randomization 1:1 to receive either a maximum total protein dose of 300 mg or 1200 mg. Week 8–18: Maintenance dose (300 mg vs. 1200 mg)	150 mg, 3 doses, every 4 weeks	8 weeks	Multiple: cashew, walnut, hazelnut, almond, sesame, cow's milk, hen's egg, peanut, soy, and/or wheat	Physician‐diagnosed FA: clinical history of allergic reaction and a positive SPT result ≥6 mm or a ImmunoCAP IgE (sIgE) >4 kU/L for each allergen	Week 0: Randomization 1:1 to receive either a maximum total protein dose of 300 mg or 1200 mg. Arm 1: Dose escalation: 5 mg/60 mg/300 mg or Arm 2: dose escalation 5 mg/240 mg/1200 mg. Week 8–18: Maintenance dose (300 mg vs. 1200 mg)	No systematic post treatment OFC	Primary: % of participants who have an increase from screening to week 18 in the ratio of food allergen sIgG4/sIgE of at least 25% for at least 2 allergens. Secondary: for at least three, four, and five allergens	Primary: 42/60 (70%) reached the primary endpoint of increase in allergen sIgG4/sIgE ratio of at least 25% at week 18, with no difference between the treatment groups (OR [95% CI] = 1.00 [0.29, 3.49]). Secondary: 24/46 (52%) for 3 allergens 8/23 (35%) for 4 allergens 2/7 (29%) for 5 allergens	No differences in AEs between the 300 and 1200 mg groups (19% vs. 17%, *p* = .69), respectively. 15% of doses associated with gastrointestinal AEs 1200 mg group: higher % of doses associated with moderate AEs compared with the 300 mg group (4% vs. 3%, *p* = .01). 3 AEs required the use of epinephrine (2 in the 300 mg group, 1 in the 200 mg group)
Badina et al.^123^	Single‐center, prospective, observational case series	*N* = 4	OMZ + CM OIT	According to s‐IgE values and weight, without exceeding the maximum recommended dose (600 mg) for asthma treatment	12 months	Cow's milk	Persisting and severe CM allergy associated with moderate allergic asthma, in which a previous OIT attempt had failed: Tolerance of ≤173 mg of CMP in open OFC	After an 8‐week course of OMZ, 2nd open OFC with CM, new OIT course maintaining cotreatment with OMZ for the first 12 months.	8 weeks (before OIT)	Threshold of CM at OFC, level of specific IgE and IgG4 for milk, QoL	All patients increased their OFC reaction threshold after 8 weeks of OMZ and maintained it long after that biologic therapy had stopped Specific milk proteins IgG4 levels significantly increased in all. QoL: improvement of outdoor life and social life, reduced anxiety and loss of fear of severe reactions due to food contaminations	All 4 patients experienced no reactions or extremely mild ones (oral itching, transient mild abdominal pain).

Abbreviations: AE, Adverse event; BAT, Basophil activation test; CM (P), cow's milk (protein); CTD, Cumulative tolerated dose; DBPCFC, double‐blind placebo‐controlled food challenge; DBRPCT, double‐blind randomized placebo‐controlled trial; EOE, Eosinophilic esophagitis; FA, Food allergy; IgE, IL, Interleukin; Immunoglobulin E; IQR, Interquartile range; OFC, Oral food challenge; OMZ, Omalizumab; QoL, Quality of Life; NOAEL, No‐observed‐adverse‐effect level; OIT, Oral Immunotherapy; WDEIA, wheat‐dependent exercise‐induced anaphylaxis.

Multi‐food OIT is an attractive OMZ‐add‐on treatment.^118^, ^119^, ^122^ In a multicenter randomized, DBPC trial by Andorf et al., OMZ treatment was initiated 8 weeks before rapid desensitization with multi‐food OIT (2–5 foods, 30 weeks, then continued to 36 weeks or decreased or discontinued). Among the 40 participants still in the active multi‐food OIT arm at week 36, 85% reached a dose of at least 2 g of at least 2 food allergens, compared with 55% in the 20 participants randomized to the OIT discontinuation arm at week 30.^119^ Some other studies have shown that OMZ combined with multi‐food OIT allows faster up‐dosing with fewer side effects compared to OIT alone.^118^, ^119^ However, current evidence does not support an additional pro‐tolerogenic effect to OIT itself.

In summary, the FDA approval of OMZ as monotherapy represents a milestone. There is hope that these options (OIT, OMZ, or both) will become available for patients with FA, expanding opportunities of new strategies for patients and families as part of shared‐decision making.^124^

#### 6.1.3 Omalizumab dosing issues

The dosing regimen of OMZ has been debated over the last few years. Many studies such as the OUTMATCH trial have used an “asthma dosing regimen” based on total IgE and weight. In this trial, higher total baseline IgE was weakly but significantly positively associated with response to OMZ.^125^ This approach has been questioned by more recent publications. In a large real‐life study of patients who underwent OMZ‐facilitated OIT, dosage based on weight alone rather than on weight and total IgE level was strongly associated with effectiveness.^126^ Comparable results have been shown by Arasi et al. in asthmatics with concomitant FA treated with OMZ.^127^ Based on this observation, a prospective DBPC trial on the dose‐related efficacy of OMZ (8 mg/kg or 16 mg/kg per month or placebo)‐facilitated poly OIT (3 foods) trial has been conducted with results to be expected shortly.^128^

### 6.2 Other monoclonal antibodies and small molecules (Table 6)

**TABLE 6:** Studies on other biologicals in food allergy.

Ref	Type of study	Patients number and age	Intervention	Dosage	Duration	Food	Reaction threshold	OIT	Timing of post‐treatment OFC	Endpoints	Results	Safety
Begin et al.^130^	Phase III, multi‐center, DBRPC study	*N* = 181	Ligelizumab	120 or 240 mg or placebo every 4 weeks	52 weeks (only 11 participants completed the full 52‐week period) Early termination of the study due to data review indicated that neither ligelizumab dose was likely to achieve 70% for the primary endpoint	Peanut	DBPCFC at screening dose‐limiting symptoms ≤100 mg (42.6% ≤3 mg)	No	Week 12 DBPCFC up to 1444 mg of peanut protein	Primary: tolerance of ≥600 mg of peanut protein after 12 weeks (comparison 120 mg/240 mg) Secondary: tolerance of higher doses of peanut protein, maximum symptom severity	Primary: 4.7% (21/47) in the 240 mg ligelizumab‐arm; 15.7% (8/51) in the 120 mg ligelizumab‐arm; 4.3% (1/23) for placebo. Secondary: reduced odds of developing more severe symptoms at week 12 in 240 mg ligelizumab‐arm vs. placebo (OR 10.59 [3.63–31–56]) and 68% of the participants in the 240 mg ligelizumab‐arm had a >10‐fold change in maximum tolerated dose	No new neg safety signals observed. No serious hypersensitivity events, such as anaphylaxis, related to ligelizumab were reported during the study
Leung et al.^97^	DB randomized dose‐ranging trial, multicenter (7 centers)	*N* = 81 12–60 years (mean: 32 years)	Talizumab (anti‐IgE)	TNX‐901150 mg (*n* = 19), 300 mg (*n* = 19), 450 mg (*n* = 21) or placebo (*n* = 23) subcutaneously every 4 weeks for four doses	16 weeks	Peanut	Mean threshold sensitivity: 330.9 mg peanut flour	No	After 20 weeks	Primary: change from baseline in the threshold dose at final OFC	Mean increases in the OFC threshold: 710 mg in the placebo group, 913 mg in the group given 150 mg of TNX‐901, 1650 mg in the group given 300 mg of TNX‐901, and 2627 mg in the group given 450 mg of TNX‐901 (*p* < .001)	Well‐tolerated: number of systemic adverse events similar between groups, mostly mild injection‐site reactions
Chinthrajah et al.^136^	Phase II, multicenter, DBRPC study	*N* = 232,148 in the Full Analysis Set Pediatric patients (6–≤17 years old), median age: 11	Dupilumab	Randomized 2:1 dupilumab + OIT vs. matching placebo + OIT every 2 weeks stratified by screening ps‐IgE level (≤ or >100 kUA/L) and body weight	52 weeks	Peanut	DBPCFC at screening: dose‐limiting symptoms ≤100 mg Mean tolerated dose: 14.8 mg (1.0–30.0 mg).	Week 4: AR101	Week 28 (post up‐dosing) Week 52 (post‐maintenance) Week 64 (end of study)	Primary: DBPCFC with 2044 mg (cumulative) of peanut protein at week 28 Secondary: DBPCFC with 2044 mg peanut protein at 52wk according to continuous treatment by Dupilumab or switch to placebo after week 28	Primary: 20.2% increase in the proportion of patients who could pass the DBPCFC to 2044 mg of peanut protein at week 28 (*p* < .05) Secondary: in patients re‐ randomized from dupilumab + OIT to placebo + OIT at week 28, the proportion who passed the DBPCFC decreased to a level similar to that observed in patients who continuously received placebo + OIT.	Good tolerance of Dupilumab, compared with placebo, dupilumab decreased the proportion of patients experiencing mild, moderate, or severe symptoms during the OIT up‐dosing phase
Emerson et al.^134^	Retrospective study	*N* = 60 6 months–18 years	Dupilumab	Treatment of atopic dermatitis	At least 6 months	Several					Greater decrease in specific IgE with longer duration if Dupilumab 0.6% (95% CI −0.8% to −0.4%, *p* < .001) for each additional month of treatment. No significant change in SPT.	
Chinthrajah et al.^139^	6‐week placebo‐controlled phase 2a study	*N* = 20 adults randomized in a 3:1 ratio 15 etokimab 5 blinded placebo	Etokimab (IgG1 anti‐IL‐33)	Single one i.v. dose of either etokimab (300 mg/100 mL) or placebo (0.9% sodium chloride [100 mL]).	6 weeks	Peanut	Positive OFC: objective reaction to ≤275 mg peanut protein.	No	OFC Day 15 Follow‐up phase: OFC on Day 45	Primary: no objective reaction to a CTD of 275 mg of peanut protein on day 15 Secondary: no objective reaction to a CTD of 275 mg of peanut protein on day 45, measurement of proinflammatory serum cytokines (IL‐4, IL‐5, IL‐9, and IL‐13), ST2, sST2, peanut‐ or histamine‐specific skin prick test wheal size, and peanut and total IgE during the study.	Primary: 73% in the etokimab vs. 0% in the Placebo group (*p* = .008) on day 15 Secondary: 57% vs. 0% (ns) with no objective reaction to a CTD of 275 mg of peanut protein on day 15 IL‐4, IL‐5, IL‐9, IL‐13, and ST2 levels in CD4^+^ T cells were reduced in the active vs. placebo arm upon peanut‐induced T cell activation (*p* = .036 for IL‐13 and IL‐9 at day 15), peanut‐specific IgE was reduced in active vs. placebo (*p* = .014 at day 15).	Most frequent AE reported in etokimab arm: headache in 4/15 (27%). Atopy‐related events (asthma, eczema, food allergy, and allergic rhinitis) in 1/15 (7%) in etokimab arm vs. 3/5 (60%) in the Placebo armp. Fewer moderate AEs in etokimab arm vs. placebo arm (60% vs. 100%). No severe AEs.

Ligelizumab, initially developed for CSU,^129^ has a higher affinity for human IgE. In a 52‐week DBPC study in peanut allergic patients, 44.7% of participants (ligelizumab 240 mg treatment arm) were able to tolerate at least 600 mg of peanut protein at week 12, compared with 4.3% in the placebo arm. However, the expected difference between the 120 mg and the 240 mg treatment dose arms was not significant, leading to the early termination of the study.^130^ Ligelizumab, despite its higher affinity for IgE, is less effective than OMZ.^131^ UB‐221 has a high affinity for IgE antibody, thereby decreasing its plasma levels, free trimeric form of the receptor, FcεRII or CD23, and the IgE‐bound form, which decreases the synthesis of IgE.^132^ A DBPC trial is currently ongoing to evaluate the safety and efficacy of UB‐221 in adults with a documented history of allergic reactions to α‐gal.^133^ Given the role of IL4 for isotype switching and effector cell activation, dupilumab in the treatment of FA has been investigated. Whereas IgE sensitization to food allergens seems to decrease for each additional month of treatment in children receiving Dupilumab for AD,^134^ there is currently no evidence of its efficacy in FA. A Phase II multicenter, open‐label study was conducted to assess dupilumab monotherapy in 24 children and adolescents with peanut allergy (reactivity to peanut ≤144 mg).^135^ The primary outcome (tolerance of at least 444 mg) was not met, with only 2 patients reaching this goal after 24 weeks of treatment. A Phase II randomized, DBPC study addressed the efficacy of dupilumab as an adjunct to peanut OIT in 123 peanut allergic children. The effect was modest with a significant but low increase (20%) in the number of patients able to tolerate at least 2044 mg of peanut protein following the up‐dosing period, compared with placebo.^136^ If the proportion of patients experiencing adverse events (including gastrointestinal symptoms) was lower in the Dupilumab + OIT group, there was no protection against OIT related anaphylaxis. An ongoing trial is studying the hypothesis that adding dupilumab to OMZ‐facilitated multi‐OIT will increase the likelihood of inducing sustained unresponsiveness and decrease OIT‐related adverse events (COMBINE study).^137^ In a pre‐clinical study (mainly in animal model), Limnander et al. investigated an interesting approach. First, a B cell maturation antigen x CD3 bispecific antibody (linvoseltamab) was used to eliminate long‐lived IgE+ plasma cells, then a second phase with the IL‐4Rα blockade (dupilumab) prevents the IL‐4‐mediated class switch to IgE, and the reemergence of IgE‐producing memory B‐cells and plasma cells, associated to allergic responses.^138^ This strategy is currently investigated in a Phase 1 trial (NCT06369467).

A phase II, DBPC study was performed to evaluate the safety and the ability of a single dose of Etokimab, an anti‐IL33 monoclonal antibody, to desensitize 20 peanut allergic adults.^139^ There was a significant increase in tolerated dose and downregulation in the number of allergen‐specific T‐cells with a T2 signature. Finally, other treatments are currently being studied, such as small molecules (remibrutinib) in adults with peanut allergies (NCT05432388), or are planned for adolescents and adults with FA.

## 7 EOSINOPHILIC GASTROINTESTINAL DISEASES

EGIDs are chronic inflammatory disorders characterized by symptoms of gastrointestinal dysfunction associated with eosinophil‐predominant infiltration of the gastrointestinal tract. EGIDs are broadly classified into EoE and non‐EoE EGIDs according to the anatomical site of inflammation.^140^, ^141^, ^142^ Although significant progress has been made in recent years, the pathophysiology of these conditions, particularly non‐EoE EGIDs, remains incompletely understood.

Evidence‐based therapeutic guidelines are currently available for EoE and include biologics.^143^, ^144^, ^145^, ^146^, ^147^, ^148^ In contrast, evidence for non‐EoE EGIDs remains limited and standardized treatment strategies have yet to be established.^140^, ^142^ Several randomized controlled trials are ongoing, with biologic agents representing a major area of investigation (Table 7).

**TABLE 7:** Biologics, small molecules and selected agents: Drug development pipeline in eosinophilic esophagitis and non‐eosinophilic esophagitis eosinophilic gastrointestinal diseases (ClinicalTrial.gov, January 2026).

Agent	Mechanism of action	Condition	Study phase	NCT number
Dupilumab	Fully human anti‐IL‐4 receptor‐α mAb that inhibits signaling from IL‐4 and IL‐13	EoE in children	3	NCT07112378^a^
Dupilumab	Fully human anti‐IL‐4 receptor‐α mAb that inhibits signaling from IL‐4 and IL‐13	Stenotic EoE in children (12–25 years old)	2	NCT06705387^a^
Solrikitug (NS‐8226)	Humanized IgG1 mAb binding TSLP	EoE in adults	2	NCT06598462^b^
Barzolvolimab (CDX‐0159)	Humanized anti‐KIT IgG1 mAb	EoE in adults	2	NCT05774184^b^
Tezepelumab	Anti‐TSLP mAb	EoE in adults and adolescents	3	NCT05583227^b^
Cendakimab (RPC4046; ABT 308; CC‐93538)	Selective, humanized, recombinant mAb targetting IL‐13	EoE in adults and adolescents	3	NCT04991935^b^
Caly − 002	Recombinant monoclonal antibody targeting interleukin‐15 (IL‐15)	Healthy Subjects and Subjects With Celiac Disease and EoE	1	NCT04593251^c^
Dupilumab	Fully human anti‐IL‐4 receptor‐α mAb that inhibits signaling from IL‐4 and IL‐13	Non EoE‐EGIDs in children	Observational	NCT07257835^a^
Dupilumab	Fully human anti‐IL‐4 receptor‐α mAb that inhibits signaling from IL‐4 and IL‐13	Non EoE EGIDs in adults and adolescents	2	NCT05831176^b^
Cendakimab (CC‐93538, RPC4046; ABT 308;CC‐93538)	Selective, humanized, recombinant mAb targetting IL‐13	EGIDs in adults and adolescents	3	NCT05214768^c^
IRL201104	Immunomodulatory peptide	EoE in adults	2a	NCT05084963^c^
Etrasimod	Selective sphingosine 1‐phosphate (S1P)_1,4,5_ receptor modulator	EoE in adults	2	NCT04682639^d^
AQ280	Selective JAK1 inhibitor	Healthy subjects	1	NCT05485779^c^

Abbreviations: EoE, eosinophilic esophagitis; Non EoE‐EGIDs, non EoE‐eosinophilic gastrointestinal diseases; mAb, monoclonal antibody; TSLP, thymic stromal lymphopoietin.

^a^ Recruiting.

^b^ Active, not recruiting.

^c^ Completed with results.

^d^ Completed.

Current recommended treatment for EoE includes proton pump inhibitors (PPI), swallowed topical corticosteroids (STC), food elimination diets (FED), and dupilumab. According to current guidelines, which may vary slightly across expert societies and national health systems, dupilumab is recommended for patients with persistent disease despite PPI^145^ or in selected cases refractory to first‐line treatment (PPI, SCT and FED), when these treatments are contraindicated, or in patients with a comorbidity for which dupilumab is already indicated.^143^, ^144^, ^146^, ^147^, ^148^, ^149^

Dupilumab was approved by the FDA in 2022 and by the EMA in 2024 for the treatment of EoE in patients aged 12 years and older weighing at least 40 kg.^150^, ^151^ In 2024, its indication was extended to children aged 1–11 years weighing at least 40 kg.^152^ Clinical trials demonstrated histologic and clinical remission rates of 60% compared with 5% with placebo at week 24, with sustained responses through week 52. Current guidelines, including those from the ESPGHAN EGID Working Group, do not recommend the routine use of other biologics; however, they may be considered within clinical trials or specialized centers pending further evidence and regulatory approval^148^ (Table 7).

Several biologics have been evaluated in EoE but failed to demonstrate meaningful clinical benefit despite eosinophil depletion. These include omalizumab, mepolizumab, benralizumab, reslizumab, and lirentelimab (an anti‐Siglec‐8 monoclonal antibody).^149^, ^153^, ^154^ Some of these agents are nevertheless being explored for non‐EoE EGIDs. In parallel, several small‐molecule therapies are under investigation for both EoE and non‐EoE EGIDs (Table 7), including the peptide immunomodulator IRL201104^155^ and etrasimod.^156^

## 8 FUTURE AND CONCLUSION

Biologics and small‐molecule treatments have significantly reshaped the therapeutic landscape of allergic diseases, enabling more personalized approaches and “treat‐to‐target” strategies. Numerous clinical trials are currently evaluating novel molecules and innovative approaches, including bispecific monoclonal antibodies targeting multiple cytokines. Pediatric trials from early childhood through adolescence are essential to address age‐specific disease characteristics and goals. In addition, prospective real‐world registries following patients from childhood into adulthood are needed to better characterize allergic multimorbidity trajectory and the transition period.

Despite these advances, important unmet needs remain. These treatments are not yet available for all pediatric allergic diseases or across all age groups, particularly in young children, even though severe allergic phenotypes may develop early in life. The identification of robust biomarkers, including those derived from omics technologies, is therefore needed to improve patient selection, predict and monitor treatment response, and guide decisions regarding discontinuation.^157^ Treatment costs also remain a major barrier, particularly in low‐income countries, although the availability of biosimilars may improve access. Finally, the development of clear clinical practice guidelines is essential to define criteria for optimal treatment selection, establish standardized outcome measures, monitor long‐term efficacy and safety, and assess the potential for treatment discontinuation. Further research is also needed to determine the long‐term impact of these treatments on disease progression and on the risk of developing additional allergic diseases in children, which may represent a key pediatric objective in the future.

## AUTHOR CONTRIBUTIONS

**Amandine Divaret Chauveau:** Writing – original draft; writing – review and editing. **Herman T. den Dekker:** Writing – original draft; writing – review and editing. **Thomas Eiwegger:** Writing – original draft; writing – review and editing. **Dorothea Holter:** Writing – original draft; writing – review and editing. **Benoit Sterling:** Writing – original draft; writing – review and editing. **Stéphanie Mallet:** Writing – original draft; writing – review and editing. **Antoine Deschildre:** Conceptualization; writing – original draft; writing – review and editing; supervision. **Stéphanie Coopman:** Writing – original draft; writing – review and editing. **R. Sharon Chinthrajah:** Writing – original draft; writing – review and editing. **Mariëlle W. Pijnenburg:** Writing – original draft; writing – review and editing. **Stéphanie Lejeune:** Writing – original draft; writing – review and editing.

## CONFLICT OF INTEREST STATEMENT

A.D.C.: reports grant from Don du Souffle, Novartis, ARAIRLOR, CICBAA, French Academy of Medicine, French Society of Pediatric Pulmonology and Allergology; speaker/consulting fees from Sanofi, Stallergenes, ALK, Aimmune Therapeutics, Novartis, DBV; support for attending meetings from Mead Johnson, Nutricia, Aimmune Therapeutics, Novartis, ALK, DBV, Stallergenes; stocks from Essilor Luxottica; Outside of the submitted work, B.S.: Abbvie, Astra Zeneca, Sanofi, Novartis, Rivadouce. S.C.: received fees from Sanofi (speaker), AstraZeneca (consulting), AbbVie (speaker, hospitality), Celltrion (hospitality), Nestlé (speaker and consulting), Thersolve (hospitality). R.S.C.: receives grant support from the Consortium for Food Allergy Research (CoFAR), National Institute of Allergy and Infectious Disease (NIAID), Food Allergy Research & Education (FARE). She is an advisory board member for Novartis, Intrommune Therapeutics, Phylaxis, Genentech, and Latitude and owns stock in Intrommune Therapeutics and Grow Happy. S.L.: has received fees for funding to participate in conferences from Sanofi and ALK, for communications from Sanofi, for training activities from Sanofi, Novartis, and Stallergènes‐Greer, and for research work from Astra Zeneca. T.E.: reports to have acted or acts as local PI for company‐sponsored trials by DBV Therapeutics, Greer Stallergens, Novartis, and sub‐investigator ALK‐Abelló. He is Co‐Investigator or scientific lead in three investigator‐initiated oral immunotherapy trials supported by the SickKids FA and Anaphylaxis Program and serves as an associate editor for Allergy. He/his lab received unconditional/in‐kind contributions from Macro Array Diagnostics and an unrestricted grant from ALK‐Abelló. He holds scientific advisory board roles for ALK‐Abelló, VAMED, Nutricia/Danone, Hipp, Sanofi, Greer‐Stallergens, Allergy Therapeutics and Aimmune. TE reports lecture fees from Novartis, ThermoFisher, Nutricia/Danone, Aimmune, Sanofi, Schwalbe, MADX, ALK‐Abelló. S.M.: received speaker/consulting fees from Lilly, Pierre Fabre, Almirall, Sanofi, Abbvie, Janssen, Léo pharma, Novartis. M.W.P.: received grants from the Netherlands Lung Fund and The Netherlands Organization for Health Research and Development; consulting fees from Sanofi, AstraZeneca and Novartis; she participates in studies of Sanofi and AstraZeneca; she is co‐chair of the European Respiratory Society Clinical research collaboration SPACE (Severe Pediatric Asthma Collaborative Europe) that has been or is funded by Novartis, AstraZeneca, Sanofi and Celltrion. A.D.: received speaker/consulting fees from Aimmune Therapeutics, ALK, AstraZeneca, Celltrion, DBV Technologies, GSK, Novartis, Regeneron Pharmaceuticals Inc., Sanofi, Stallergenes Greer, Viatris – outside the submitted work. He is a member of the steering committee of the European Respiratory Society Clinical research collaboration SPACE (Severe Pediatric Asthma Collaborative Europe) that has been or is funded by Novartis, AstraZeneca, Sanofi, and Celltrion. H.T.d.D., D.H.: none to declare.

## Data Availability

Data sharing not applicable to this article as no datasets were generated or analysed during the current study.
